# Supplementary material for: Ecology, more than antibiotics consumption, is the major predictor for the global distribution of aminoglycoside-modifying enzymes
Source: eLife. 2023 Feb 14;12:e77015. doi: 10.7554/eLife.77015 (PMC9928423; doi:10.7554/eLife.77015)
Supplement: Supplementary file 2. [file elife-77015-supp2.doc]

**Supplementary file 2a: Summary of the selected model for AACa for worldwide data.** *: p<0.05, **: p<0.01, ***: p<0.001.

| Autoregression coefficient φ = 0.264, Matérn smoothness ν = 16.7, Matérn scaling ρ = 0.729 | | | | |
| --- | --- | --- | --- | --- |
| **Variable** | **Estimate** | **Conditional standard error** | **t** | **p** |
| Intercept | -7.91 | 0.833 | -9.51 | 0 *** |
| Domestic animals | -29.8 | 4.08 × 10^6^ | -7.32 × 10^-6^ | 1 |
| Farms | 0.411 | 0.76 | 0.541 | 0.589 |
| Flora, fauna | -29.9 | 3.97 × 10^6^ | -7.54 × 10^-6^ | 1 |
| Human samples | -0.307 | 0.815 | -0.376 | 0.707 |
| Human habitat | 0.279 | 1.24 | 0.225 | 0.822 |
| Freshwater | -0.622 | 1.23 | -0.504 | 0.614 |
| Clinical samples | 1.65 | 0.721 | 2.28 | 2.23 × 10^-2^ * |
| Sludge, waste | 2.2 | 0.933 | 2.35 | 1.86 × 10^-2^ * |
| Soil | -30.1 | 2.02 × 10^6^ | -1.49 × 10^-5^ | 1 |
| Trade | -0.136 | 7.04 × 10^-2^ | -1.93 | 5.36 × 10^-2^ * |
| Migration | -0.162 | 7.78 × 10^-2^ | -2.09 | 3.68 × 10^-2^ * |

**Supplementary file 2b: Summary of the selected model for AACb for worldwide data.** *: p<0.05, **: p<0.01, ***: p<0.001.

| Autoregression coefficient φ = -0.167, Matérn smoothness ν = 9.1 × 10^-3^, Matérn scaling ρ = 1.48 | | | | |
| --- | --- | --- | --- | --- |
| **Variable** | **Estimate** | **Conditional standard error** | **t** | **p** |
| Intercept | -5.81 | 0.556 | -10.4 | 0 *** |
| Domestic animals | -32 | 4.43 × 10^6^ | -7.22 × 10^-6^ | 1 |
| Farms | -0.117 | 0.277 | -0.423 | 0.673 |
| Flora, fauna | -20.2 | 23.9 | -0.846 | 0.397 |
| Human samples | -2.84 | 0.657 | -4.32 | 1.57 × 10^-5^ *** |
| Human habitat | -31.6 | 4.08 × 10^6^ | -7.74 × 10^-6^ | 1 |
| Freshwater | -3.6 | 1.68 | -2.15 | 3.17 × 10^-2^ * |
| Clinical samples | -2.04 | 0.282 | -7.26 | 3.98 × 10^-13^ *** |
| Sludge, waste | -31.1 | 3.88 × 10^6^ | -8 × 10^-6^ | 1 |
| Soil | -1.4 | 0.588 | -2.38 | 1.73 × 10^-2^ * |
| Trade | -1.03 | 0.317 | -3.26 | 1.11 × 10^-3^ ** |
| Migration | 1.09 | 0.168 | 6.5 | 8.08 × 10^-11^ *** |
| Domestic animals × Trade | 0.743 | 2.22 × 10^6^ | 3.35 × 10^-7^ | 1 |
| Farms × Trade | 0.95 | 0.307 | 3.1 | 1.97 × 10^-3^ ** |
| Flora, fauna × Trade | -83.2 | 87.9 | -0.947 | 0.343 |
| Human samples × Trade | -0.455 | 1.78 | -0.256 | 0.798 |
| Human habitat × Trade | 0.741 | 1.17 × 10^6^ | 6.33 × 10^-7^ | 1 |
| Freshwater × Trade | -7.78 | 5.67 | -1.37 | 0.17 |
| Clinical samples × Trade | 0.958 | 0.31 | 3.09 | 2.03 × 10^-3^ ** |
| Sludge, waste × Trade | 0.618 | 3.11 × 10^6^ | 1.99 × 10^-7^ | 1 |
| Soil × Trade | 0.781 | 0.566 | 1.38 | 0.167 |
| Domestic animals × Migration | -1.01 | 2.19 × 10^6^ | -4.62 × 10^-7^ | 1 |
| Farms × Migration | -1.33 | 0.216 | -6.15 | 7.85 × 10^-10^ *** |
| Flora, fauna × Migration | -0.18 | 2.61 | -6.88 × 10^-2^ | 0.945 |
| Human samples × Migration | -2.09 | 1.46 | -1.43 | 0.154 |
| Human habitat × Migration | -1.46 | 4.82 × 10^6^ | -3.03 × 10^-7^ | 1 |
| Freshwater × Migration | -3.82 | 4.09 | -0.932 | 0.351 |
| Clinical samples × Migration | -1.61 | 0.313 | -5.13 | 2.84 × 10^-7^ *** |
| Sludge, waste × Migration | -1.28 | 3.76 × 10^6^ | -3.41 × 10^-7^ | 1 |
| Soil × Migration | -1.21 | 0.856 | -1.41 | 0.158 |

**Supplementary file 2c: Summary of the selected model for AACc for worldwide data.** *: p<0.05, **: p<0.01, ***: p<0.001.

| Autoregression coefficient φ = -0.208, Matérn smoothness ν = 1.53, Matérn scaling ρ = 2.18 | | | | |
| --- | --- | --- | --- | --- |
| **Variable** | **Estimate** | **Conditional standard error** | **t** | **p** |
| Intercept | -8.37 | 0.984 | -8.51 | 0 *** |
| Domestic animals | -1.94 × 10^3^ | 2.9 × 10^5^ | -6.71 × 10^-3^ | 0.995 |
| Farms | -1.59 | 1.44 | -1.1 | 0.27 |
| Flora, fauna | 2.83 | 1.12 | 2.54 | 1.12 × 10^-2^ * |
| Human samples | 2.92 | 0.857 | 3.4 | 6.64 × 10^-4^ *** |
| Human habitat | 0.778 | 1.57 | 0.495 | 0.621 |
| Freshwater | 3.3 × 10^-2^ | 1.15 | 2.85 × 10^-2^ | 0.977 |
| Clinical samples | 3.48 | 0.853 | 4.08 | 4.59 × 10^-5^ *** |
| Sludge, waste | -1.08 | 2.33 | -0.464 | 0.643 |
| Soil | 2.36 | 0.888 | 2.66 | 7.88 × 10^-3^ ** |
| Trade | -0.7 | 0.429 | -1.63 | 0.103 |
| Migration | -1.41 | 1.63 | -0.864 | 0.387 |
| Domestic animals × Trade | 2.14 × 10^2^ | 3.2 × 10^4^ | 6.67 × 10^-3^ | 0.995 |
| Farms × Trade | -3.17 | 2.17 | -1.46 | 0.143 |
| Flora, fauna × Trade | -1.18 | 1.49 | -0.793 | 0.428 |
| Human samples × Trade | -0.76 | 0.449 | -1.69 | 9.08 × 10^-2^ * |
| Human habitat × Trade | 9.91 × 10^-2^ | 0.992 | 0.1 | 0.92 |
| Freshwater × Trade | -0.7 | 0.512 | -1.37 | 0.172 |
| Clinical samples × Trade | 0.495 | 0.429 | 1.15 | 0.249 |
| Sludge, waste × Trade | -4.15 | 3.48 | -1.19 | 0.233 |
| Soil × Trade | 0.743 | 0.453 | 1.64 | 0.1 |
| Domestic animals × Migration | -2.94 × 10^3^ | 4.37 × 10^5^ | -6.72 × 10^-3^ | 0.995 |
| Farms × Migration | -0.703 | 2.21 | -0.318 | 0.75 |
| Flora, fauna × Migration | 0.675 | 1.72 | 0.392 | 0.695 |
| Human samples × Migration | 1.55 | 1.63 | 0.95 | 0.342 |
| Human habitat × Migration | -5.3 × 10^-2^ | 2.47 | -2.14 × 10^-2^ | 0.983 |
| Freshwater × Migration | -6.51 | 2.14 | -3.04 | 2.4 × 10^-3^ ** |
| Clinical samples × Migration | 1.44 | 1.63 | 0.882 | 0.378 |
| Sludge, waste × Migration | -4.59 | 5.36 | -0.857 | 0.391 |
| Soil × Migration | 7.08 × 10^-2^ | 1.67 | 4.25 × 10^-2^ | 0.966 |

**Supplementary file 2d: Summary of the selected model for AACd for worldwide data.** *: p<0.05, **: p<0.01, ***: p<0.001.

| Autoregression coefficient φ = 0.216, Matérn smoothness ν = 5.49 × 10^-3^, Matérn scaling ρ = 2.12 | | | | |
| --- | --- | --- | --- | --- |
| **Variable** | **Estimate** | **Conditional standard error** | **t** | **p** |
| Intercept | -6.84 | 0.698 | -9.81 | 0 *** |
| Domestic animals | -30.7 | 4.59 × 10^6^ | -6.69 × 10^-6^ | 1 |
| Farms | 0.109 | 0.49 | 0.222 | 0.824 |
| Flora, fauna | 2.27 | 0.598 | 3.79 | 1.48 × 10^-4^ *** |
| Human samples | -1.99 | 0.956 | -2.08 | 3.71 × 10^-2^ * |
| Human habitat | -3.12 | 1.62 | -1.93 | 5.37 × 10^-2^ * |
| Freshwater | -3.28 | 3.4 | -0.965 | 0.335 |
| Clinical samples | -0.106 | 0.459 | -0.231 | 0.817 |
| Sludge, waste | -31.2 | 5.99 × 10^6^ | -5.21 × 10^-6^ | 1 |
| Soil | -1.45 | 1.24 | -1.17 | 0.243 |
| Trade | 0.328 | 0.437 | 0.752 | 0.452 |
| Migration | -1.8 | 0.983 | -1.83 | 6.7 × 10^-2^ * |
| Domestic animals × Trade | -1.12 | 6.93 × 10^6^ | -1.62 × 10^-7^ | 1 |
| Farms × Trade | -1.59 | 0.527 | -3.01 | 2.6 × 10^-3^ ** |
| Flora, fauna × Trade | -2.47 | 1.16 | -2.13 | 3.32 × 10^-2^ * |
| Human samples × Trade | -1.03 | 0.741 | -1.39 | 0.163 |
| Human habitat × Trade | 0.215 | 0.973 | 0.221 | 0.825 |
| Freshwater × Trade | -16.4 | 14.4 | -1.14 | 0.254 |
| Clinical samples × Trade | -0.478 | 0.44 | -1.09 | 0.277 |
| Sludge, waste × Trade | -0.682 | 5.94 × 10^6^ | -1.15 × 10^-7^ | 1 |
| Soil × Trade | -0.349 | 1.21 | -0.288 | 0.774 |
| Domestic animals × Migration | 2.23 | 7.94 × 10^6^ | 2.81 × 10^-7^ | 1 |
| Farms × Migration | 0.544 | 1.11 | 0.488 | 0.625 |
| Flora, fauna × Migration | 1.62 | 1.24 | 1.31 | 0.191 |
| Human samples × Migration | -3.29 | 3.33 | -0.986 | 0.324 |
| Human habitat × Migration | -9.21 | 6.46 | -1.43 | 0.154 |
| Freshwater × Migration | -1.16 | 6.12 | -0.189 | 0.85 |
| Clinical samples × Migration | 1.42 | 0.985 | 1.44 | 0.15 |
| Sludge, waste × Migration | 1.82 | 6.44 × 10^6^ | 2.83 × 10^-7^ | 1 |
| Soil × Migration | 1.22 | 1.8 | 0.68 | 0.496 |

**Supplementary file 2e: Summary of the selected model for AACe1 for worldwide data.** *: p<0.05, **: p<0.01, ***: p<0.001.

| Autoregression coefficient φ = 0.755, Matérn smoothness ν = 1.27 × 10^-2^, Matérn scaling ρ = 1.17 | | | | |
| --- | --- | --- | --- | --- |
| **Variable** | **Estimate** | **Conditional standard error** | **t** | **p** |
| Intercept | -1.43 × 10^2^ | 6.01 × 10^3^ | -2.38 × 10^-2^ | 0.981 |
| Domestic animals | 1.33 × 10^2^ | 6.01 × 10^3^ | 2.22 × 10^-2^ | 0.982 |
| Farms | 1.35 × 10^2^ | 6.01 × 10^3^ | 2.25 × 10^-2^ | 0.982 |
| Flora, fauna | 1.38 × 10^2^ | 6.01 × 10^3^ | 2.3 × 10^-2^ | 0.982 |
| Human samples | 1.37 × 10^2^ | 6.01 × 10^3^ | 2.28 × 10^-2^ | 0.982 |
| Human habitat | 1.37 × 10^2^ | 6.01 × 10^3^ | 2.29 × 10^-2^ | 0.982 |
| Freshwater | -1.55 × 10^2^ | 6.83 × 10^4^ | -2.28 × 10^-3^ | 0.998 |
| Clinical samples | 1.39 × 10^2^ | 6.01 × 10^3^ | 2.31 × 10^-2^ | 0.982 |
| Sludge, waste | 1.39 × 10^2^ | 6.01 × 10^3^ | 2.31 × 10^-2^ | 0.982 |
| Soil | 1.32 × 10^2^ | 6.01 × 10^3^ | 2.2 × 10^-2^ | 0.982 |
| Trade | 32.5 | 1.43 × 10^3^ | 2.27 × 10^-2^ | 0.982 |
| Migration | -7.17 | 3.77 × 10^2^ | -1.9 × 10^-2^ | 0.985 |
| Domestic animals × Trade | -31.3 | 1.43 × 10^3^ | -2.18 × 10^-2^ | 0.983 |
| Farms × Trade | -31.8 | 1.43 × 10^3^ | -2.22 × 10^-2^ | 0.982 |
| Flora, fauna × Trade | -33.2 | 1.43 × 10^3^ | -2.32 × 10^-2^ | 0.982 |
| Human samples × Trade | -31.7 | 1.43 × 10^3^ | -2.21 × 10^-2^ | 0.982 |
| Human habitat × Trade | -32.6 | 1.43 × 10^3^ | -2.28 × 10^-2^ | 0.982 |
| Freshwater × Trade | 33.7 | 1.54 × 10^4^ | 2.19 × 10^-3^ | 0.998 |
| Clinical samples × Trade | -32.9 | 1.43 × 10^3^ | -2.29 × 10^-2^ | 0.982 |
| Sludge, waste × Trade | -32.6 | 1.43 × 10^3^ | -2.27 × 10^-2^ | 0.982 |
| Soil × Trade | -32 | 1.43 × 10^3^ | -2.24 × 10^-2^ | 0.982 |
| Domestic animals × Migration | 6.77 | 3.77 × 10^2^ | 1.8 × 10^-2^ | 0.986 |
| Farms × Migration | 6.55 | 3.77 × 10^2^ | 1.74 × 10^-2^ | 0.986 |
| Flora, fauna × Migration | 6.58 | 3.77 × 10^2^ | 1.75 × 10^-2^ | 0.986 |
| Human samples × Migration | 6.48 | 3.77 × 10^2^ | 1.72 × 10^-2^ | 0.986 |
| Human habitat × Migration | 6.99 | 3.77 × 10^2^ | 1.85 × 10^-2^ | 0.985 |
| Freshwater × Migration | -4.24 × 10^2^ | 1.01 × 10^5^ | -4.18 × 10^-3^ | 0.997 |
| Clinical samples × Migration | 7.33 | 3.77 × 10^2^ | 1.94 × 10^-2^ | 0.984 |
| Sludge, waste × Migration | 6.91 | 3.77 × 10^2^ | 1.83 × 10^-2^ | 0.985 |
| Soil × Migration | 1.92 | 3.77 × 10^2^ | 5.09 × 10^-3^ | 0.996 |

**Supplementary file 2f: Summary of the selected model for AACf1 for worldwide data.** *: p<0.05, **: p<0.01, ***: p<0.001.

| Autoregression coefficient φ = 0.747, Matérn smoothness ν = 0.415, Matérn scaling ρ = 4.37 × 10^-2^ | | | | |
| --- | --- | --- | --- | --- |
| **Variable** | **Estimate** | **Conditional standard error** | **t** | **p** |
| Intercept | -3.24 | 0.718 | -4.51 | 6.49 × 10^-6^ *** |
| Domestic animals | -1.2 | 0.417 | -2.88 | 3.94 × 10^-3^ ** |
| Farms | 1.03 | 0.134 | 7.71 | 1.28 × 10^-14^ *** |
| Flora, fauna | 1.31 | 0.247 | 5.3 | 1.13 × 10^-7^ *** |
| Human samples | 0.5 | 0.133 | 3.75 | 1.77 × 10^-4^ *** |
| Human habitat | 0.329 | 0.269 | 1.22 | 0.221 |
| Freshwater | -0.541 | 0.287 | -1.88 | 5.96 × 10^-2^ * |
| Clinical samples | -1.25 | 0.133 | -9.34 | 0 *** |
| Sludge, waste | -0.25 | 0.419 | -0.597 | 0.551 |
| Soil | -1.56 | 0.2 | -7.81 | 5.55 × 10^-15^ *** |
| Trade | -0.131 | 8.09 × 10^-2^ | -1.62 | 0.105 |
| Migration | -0.679 | 0.121 | -5.63 | 1.77 × 10^-8^ *** |
| Domestic animals × Trade | -0.293 | 0.326 | -0.897 | 0.37 |
| Farms × Trade | -0.531 | 8.33 × 10^-2^ | -6.38 | 1.73 × 10^-10^ *** |
| Flora, fauna × Trade | -2.51 | 0.492 | -5.09 | 3.5 × 10^-7^ *** |
| Human samples × Trade | -0.981 | 9.27 × 10^-2^ | -10.6 | 0 *** |
| Human habitat × Trade | -0.654 | 0.227 | -2.87 | 4.04 × 10^-3^ ** |
| Freshwater × Trade | -0.783 | 0.131 | -5.96 | 2.6 × 10^-9^ *** |
| Clinical samples × Trade | -0.224 | 8.33 × 10^-2^ | -2.68 | 7.3 × 10^-3^ ** |
| Sludge, waste × Trade | -0.622 | 0.415 | -1.5 | 0.134 |
| Soil × Trade | 0.19 | 0.128 | 1.48 | 0.14 |
| Domestic animals × Migration | 0.102 | 0.342 | 0.3 | 0.764 |
| Farms × Migration | 0.179 | 0.116 | 1.55 | 0.121 |
| Flora, fauna × Migration | 0.26 | 0.25 | 1.04 | 0.299 |
| Human samples × Migration | 0.867 | 0.116 | 7.47 | 8.17 × 10^-14^ *** |
| Human habitat × Migration | 0.833 | 0.222 | 3.76 | 1.72 × 10^-4^ *** |
| Freshwater × Migration | -3.19 | 0.416 | -7.68 | 1.63 × 10^-14^ *** |
| Clinical samples × Migration | 0.362 | 0.114 | 3.18 | 1.49 × 10^-3^ ** |
| Sludge, waste × Migration | 0.692 | 0.251 | 2.76 | 5.72 × 10^-3^ ** |
| Soil × Migration | -0.409 | 0.239 | -1.71 | 8.77 × 10^-2^ * |

**Supplementary file 2g: Summary of the selected model for AACg for worldwide data.** *: p<0.05, **: p<0.01, ***: p<0.001.

| Autoregression coefficient φ = -0.599, Matérn smoothness ν = 16.7, Matérn scaling ρ = 1.51 | | | | |
| --- | --- | --- | --- | --- |
| **Variable** | **Estimate** | **Conditional standard error** | **t** | **p** |
| Intercept | -4.9 | 0.437 | -11.2 | 0 *** |
| Domestic animals | 0.697 | 0.575 | 1.21 | 0.225 |
| Farms | 0.253 | 0.308 | 0.822 | 0.411 |
| Flora, fauna | 1.23 | 0.487 | 2.52 | 1.17 × 10^-2^ * |
| Human samples | 0.697 | 0.292 | 2.38 | 1.71 × 10^-2^ * |
| Human habitat | 0.398 | 0.501 | 0.796 | 0.426 |
| Freshwater | -0.776 | 0.592 | -1.31 | 0.19 |
| Clinical samples | 0.785 | 0.278 | 2.83 | 4.71 × 10^-3^ ** |
| Sludge, waste | -1.03 | 1.6 | -0.642 | 0.521 |
| Soil | 0.321 | 0.451 | 0.711 | 0.477 |
| Trade | -0.111 | 0.257 | -0.434 | 0.664 |
| Migration | 0.182 | 0.145 | 1.25 | 0.21 |
| Domestic animals × Trade | 6.37 × 10^-2^ | 0.43 | 0.148 | 0.882 |
| Farms × Trade | 0.229 | 0.267 | 0.857 | 0.391 |
| Flora, fauna × Trade | -1.88 | 1.16 | -1.62 | 0.105 |
| Human samples × Trade | 0.554 | 0.258 | 2.15 | 3.17 × 10^-2^ * |
| Human habitat × Trade | -0.434 | 0.756 | -0.575 | 0.565 |
| Freshwater × Trade | -0.488 | 0.884 | -0.552 | 0.581 |
| Clinical samples × Trade | -2.93 × 10^-3^ | 0.259 | -1.13 × 10^-2^ | 0.991 |
| Sludge, waste × Trade | -3.69 | 4.33 | -0.852 | 0.394 |
| Soil × Trade | 0.593 | 0.287 | 2.06 | 3.9 × 10^-2^ * |
| Domestic animals × Migration | -0.343 | 0.268 | -1.28 | 0.2 |
| Farms × Migration | -0.714 | 0.188 | -3.79 | 1.5 × 10^-4^ *** |
| Flora, fauna × Migration | -0.519 | 0.578 | -0.897 | 0.37 |
| Human samples × Migration | -0.383 | 0.154 | -2.49 | 1.28 × 10^-2^ * |
| Human habitat × Migration | 0.946 | 0.479 | 1.98 | 4.83 × 10^-2^ * |
| Freshwater × Migration | -0.174 | 0.657 | -0.265 | 0.791 |
| Clinical samples × Migration | -0.376 | 0.147 | -2.55 | 1.09 × 10^-2^ * |
| Sludge, waste × Migration | 1.43 × 10^-2^ | 1.77 | 8.05 × 10^-3^ | 0.994 |
| Soil × Migration | -1.93 | 0.747 | -2.58 | 9.84 × 10^-3^ ** |

**Supplementary file 2h: Summary of the selected model for ANTa for worldwide data.** *: p<0.05, **: p<0.01, ***: p<0.001.

| Autoregression coefficient φ = 0.28, Matérn smoothness ν = 16.7, Matérn scaling ρ = 0.456 | | | | |
| --- | --- | --- | --- | --- |
| **Variable** | **Estimate** | **Conditional standard error** | **t** | **p** |
| Intercept | -5.16 | 0.519 | -9.94 | 0 *** |
| Domestic animals | 0.615 | 0.519 | 1.18 | 0.236 |
| Farms | 0.941 | 0.274 | 3.43 | 5.98 × 10^-4^ *** |
| Flora, fauna | 0.265 | 0.538 | 0.493 | 0.622 |
| Human samples | -2.76 × 10^-2^ | 0.28 | -9.88 × 10^-2^ | 0.921 |
| Human habitat | 0.471 | 0.396 | 1.19 | 0.234 |
| Freshwater | 0.299 | 0.569 | 0.526 | 0.599 |
| Clinical samples | 0.303 | 0.264 | 1.15 | 0.251 |
| Sludge, waste | -3.42 | 2.59 | -1.32 | 0.186 |
| Soil | 0.759 | 0.332 | 2.29 | 2.22 × 10^-2^ * |
| Trade | -4.04 × 10^-3^ | 0.223 | -1.81 × 10^-2^ | 0.986 |
| Migration | -0.106 | 0.221 | -0.48 | 0.631 |
| Domestic animals × Trade | -0.799 | 0.616 | -1.3 | 0.195 |
| Farms × Trade | -0.482 | 0.24 | -2.01 | 4.45 × 10^-2^ * |
| Flora, fauna × Trade | 3.12 × 10^-2^ | 0.503 | 6.21 × 10^-2^ | 0.95 |
| Human samples × Trade | 0.299 | 0.227 | 1.32 | 0.188 |
| Human habitat × Trade | 0.634 | 0.275 | 2.31 | 2.12 × 10^-2^ * |
| Freshwater × Trade | -0.781 | 0.401 | -1.95 | 5.14 × 10^-2^ * |
| Clinical samples × Trade | -0.201 | 0.225 | -0.895 | 0.371 |
| Sludge, waste × Trade | 0.237 | 1.39 | 0.171 | 0.864 |
| Soil × Trade | 2.5 × 10^-2^ | 0.277 | 9.03 × 10^-2^ | 0.928 |
| Domestic animals × Migration | -0.199 | 0.417 | -0.478 | 0.633 |
| Farms × Migration | -0.929 | 0.251 | -3.7 | 2.17 × 10^-4^ *** |
| Flora, fauna × Migration | -6.91 × 10^-2^ | 0.428 | -0.161 | 0.872 |
| Human samples × Migration | -5.02 × 10^-2^ | 0.223 | -0.225 | 0.822 |
| Human habitat × Migration | -0.292 | 0.278 | -1.05 | 0.293 |
| Freshwater × Migration | -2.76 | 1.03 | -2.68 | 7.42 × 10^-3^ ** |
| Clinical samples × Migration | -0.371 | 0.22 | -1.69 | 9.12 × 10^-2^ * |
| Sludge, waste × Migration | 0.144 | 0.992 | 0.145 | 0.885 |
| Soil × Migration | -0.265 | 0.285 | -0.928 | 0.353 |

**Supplementary file 2i: Summary of the selected model for ANTb for worldwide data.** *: p<0.05, **: p<0.01, ***: p<0.001.

| Autoregression coefficient φ = 0.671, Matérn smoothness ν = 16.7, Matérn scaling ρ = 0.345 | | | | |
| --- | --- | --- | --- | --- |
| **Variable** | **Estimate** | **Conditional standard error** | **t** | **p** |
| Intercept | -10.1 | 2.03 | -4.96 | 7.17 × 10^-7^ *** |
| Domestic animals | -2.74 | 4.7 | -0.581 | 0.561 |
| Farms | 2.9 | 1.84 | 1.57 | 0.116 |
| Flora, fauna | 1.18 | 2.48 | 0.477 | 0.633 |
| Human samples | 3.56 | 1.84 | 1.94 | 5.27 × 10^-2^ * |
| Human habitat | -16.9 | 17.1 | -0.99 | 0.322 |
| Freshwater | 1.77 | 1.95 | 0.908 | 0.364 |
| Clinical samples | 3.51 | 1.84 | 1.91 | 5.62 × 10^-2^ * |
| Sludge, waste | -28 | 5.67 × 10^6^ | -4.93 × 10^-6^ | 1 |
| Soil | 2.39 | 1.9 | 1.26 | 0.208 |
| Trade | -0.168 | 0.4 | -0.42 | 0.675 |
| Migration | -4.12 | 4.49 | -0.918 | 0.359 |
| Domestic animals × Trade | -7.07 | 5.47 | -1.29 | 0.196 |
| Farms × Trade | -0.644 | 0.429 | -1.5 | 0.133 |
| Flora, fauna × Trade | 0.665 | 0.764 | 0.871 | 0.384 |
| Human samples × Trade | 0.281 | 0.4 | 0.702 | 0.482 |
| Human habitat × Trade | -2.31 | 5.16 | -0.448 | 0.654 |
| Freshwater × Trade | -0.69 | 0.736 | -0.937 | 0.349 |
| Clinical samples × Trade | -8.78 × 10^-2^ | 0.399 | -0.22 | 0.826 |
| Sludge, waste × Trade | -0.4 | 5.77 × 10^6^ | -6.94 × 10^-8^ | 1 |
| Soil × Trade | 7.43 × 10^-2^ | 0.453 | 0.164 | 0.87 |
| Domestic animals × Migration | -5.16 | 12.2 | -0.422 | 0.673 |
| Farms × Migration | 4.43 | 4.49 | 0.986 | 0.324 |
| Flora, fauna × Migration | 2.4 | 5.45 | 0.441 | 0.659 |
| Human samples × Migration | 4.18 | 4.49 | 0.93 | 0.352 |
| Human habitat × Migration | -35.7 | 35.5 | -1.01 | 0.315 |
| Freshwater × Migration | 4.95 | 4.52 | 1.09 | 0.274 |
| Clinical samples × Migration | 3.65 | 4.49 | 0.814 | 0.416 |
| Sludge, waste × Migration | 4.59 | 7.67 × 10^6^ | 5.98 × 10^-7^ | 1 |
| Soil × Migration | 3.66 | 4.58 | 0.8 | 0.424 |

**Supplementary file 2j: Summary of the selected model for ANTd for worldwide data.** *: p<0.05, **: p<0.01, ***: p<0.001.

| Autoregression coefficient φ = 0.914, Matérn smoothness ν = 6.33 × 10^-3^, Matérn scaling ρ = 0.303 | | | | |
| --- | --- | --- | --- | --- |
| **Variable** | **Estimate** | **Conditional standard error** | **t** | **p** |
| Intercept | -24.5 | 3.48 × 10^3^ | -7.03 × 10^-3^ | 0.994 |
| Domestic animals | -2.15 × 10^2^ | 2.63 × 10^6^ | -8.16 × 10^-5^ | 1 |
| Farms | 17.3 | 3.48 × 10^3^ | 4.97 × 10^-3^ | 0.996 |
| Flora, fauna | 17.1 | 3.48 × 10^3^ | 4.91 × 10^-3^ | 0.996 |
| Human samples | 18.4 | 3.48 × 10^3^ | 5.29 × 10^-3^ | 0.996 |
| Human habitat | 3.44 | 3.48 × 10^3^ | 9.88 × 10^-4^ | 0.999 |
| Freshwater | -2.74 × 10^2^ | 2.43 × 10^6^ | -1.13 × 10^-4^ | 1 |
| Clinical samples | 18.6 | 3.48 × 10^3^ | 5.34 × 10^-3^ | 0.996 |
| Sludge, waste | 19.9 | 3.48 × 10^3^ | 5.73 × 10^-3^ | 0.995 |
| Soil | 7.25 | 3.48 × 10^3^ | 2.08 × 10^-3^ | 0.998 |
| Trade | -0.847 | 3.85 × 10^3^ | -2.2 × 10^-4^ | 1 |
| Migration | -0.154 | 2.48 × 10^3^ | -6.2 × 10^-5^ | 1 |
| Domestic animals × Trade | -3.48 × 10^2^ | 2.26 × 10^6^ | -1.54 × 10^-4^ | 1 |
| Farms × Trade | -0.535 | 3.85 × 10^3^ | -1.39 × 10^-4^ | 1 |
| Flora, fauna × Trade | -1.36 | 3.85 × 10^3^ | -3.54 × 10^-4^ | 1 |
| Human samples × Trade | 0.598 | 3.85 × 10^3^ | 1.55 × 10^-4^ | 1 |
| Human habitat × Trade | 0.8 | 3.85 × 10^3^ | 2.08 × 10^-4^ | 1 |
| Freshwater × Trade | 4.81 | 2.59 × 10^6^ | 1.86 × 10^-6^ | 1 |
| Clinical samples × Trade | 9.19 × 10^-2^ | 3.85 × 10^3^ | 2.39 × 10^-5^ | 1 |
| Sludge, waste × Trade | -0.213 | 3.85 × 10^3^ | -5.53 × 10^-5^ | 1 |
| Soil × Trade | 1.3 | 3.85 × 10^3^ | 3.37 × 10^-4^ | 1 |
| Domestic animals × Migration | 1.36 × 10^2^ | 1.69 × 10^6^ | 8.07 × 10^-5^ | 1 |
| Farms × Migration | -3.3 | 2.48 × 10^3^ | -1.33 × 10^-3^ | 0.999 |
| Flora, fauna × Migration | -3.74 | 2.48 × 10^3^ | -1.51 × 10^-3^ | 0.999 |
| Human samples × Migration | 0.108 | 2.48 × 10^3^ | 4.36 × 10^-5^ | 1 |
| Human habitat × Migration | -48.6 | 2.48 × 10^3^ | -1.96 × 10^-2^ | 0.984 |
| Freshwater × Migration | 1.16 | 2.65 × 10^6^ | 4.39 × 10^-7^ | 1 |
| Clinical samples × Migration | 0.192 | 2.48 × 10^3^ | 7.71 × 10^-5^ | 1 |
| Sludge, waste × Migration | -0.161 | 2.48 × 10^3^ | -6.47 × 10^-5^ | 1 |
| Soil × Migration | -31.6 | 2.48 × 10^3^ | -1.27 × 10^-2^ | 0.99 |

**Supplementary file 2k: Summary of the selected model for APHa for worldwide data.** *: p<0.05, **: p<0.01, ***: p<0.001.

| Autoregression coefficient φ = 0.294, Matérn smoothness ν = 16.7, Matérn scaling ρ = 0.922 | | | | |
| --- | --- | --- | --- | --- |
| **Variable** | **Estimate** | **Conditional standard error** | **t** | **p** |
| Intercept | -8.94 | 1.92 | -4.66 | 3.23 × 10^-6^ *** |
| Domestic animals | -29 | 4.39 × 10^6^ | -6.6 × 10^-6^ | 1 |
| Farms | 2.93 | 1.87 | 1.57 | 0.117 |
| Flora, fauna | 0.685 | 3.3 | 0.207 | 0.836 |
| Human samples | -5.35 | 3.29 | -1.63 | 0.104 |
| Human habitat | -28.4 | 3.88 × 10^6^ | -7.33 × 10^-6^ | 1 |
| Freshwater | -28.9 | 2.55 × 10^6^ | -1.13 × 10^-5^ | 1 |
| Clinical samples | 1.11 | 1.87 | 0.591 | 0.555 |
| Sludge, waste | -29.2 | 5.48 × 10^6^ | -5.32 × 10^-6^ | 1 |
| Soil | -28.4 | 2.13 × 10^6^ | -1.34 × 10^-5^ | 1 |
| Trade | -0.984 | 1.55 | -0.635 | 0.526 |
| Migration | -1.73 | 6.3 | -0.275 | 0.783 |
| Domestic animals × Trade | 0.468 | 2.76 × 10^6^ | 1.69 × 10^-7^ | 1 |
| Farms × Trade | 0.83 | 1.55 | 0.537 | 0.591 |
| Flora, fauna × Trade | -0.228 | 3.42 | -6.67 × 10^-2^ | 0.947 |
| Human samples × Trade | 1.27 | 1.57 | 0.806 | 0.42 |
| Human habitat × Trade | 0.426 | 2.01 × 10^6^ | 2.12 × 10^-7^ | 1 |
| Freshwater × Trade | 0.504 | 1.44 × 10^6^ | 3.5 × 10^-7^ | 1 |
| Clinical samples × Trade | 0.269 | 1.56 | 0.173 | 0.862 |
| Sludge, waste × Trade | 0.452 | 2.4 × 10^6^ | 1.88 × 10^-7^ | 1 |
| Soil × Trade | 0.197 | 2 × 10^6^ | 9.86 × 10^-8^ | 1 |
| Domestic animals × Migration | 1.63 | 2.67 × 10^6^ | 6.11 × 10^-7^ | 1 |
| Farms × Migration | 1.76 | 6.3 | 0.28 | 0.78 |
| Flora, fauna × Migration | -0.181 | 12 | -1.5 × 10^-2^ | 0.988 |
| Human samples × Migration | -21 | 10.3 | -2.05 | 4.08 × 10^-2^ * |
| Human habitat × Migration | 1.38 | 5.88 × 10^6^ | 2.34 × 10^-7^ | 1 |
| Freshwater × Migration | 1.36 | 3.21 × 10^6^ | 4.25 × 10^-7^ | 1 |
| Clinical samples × Migration | 1.4 | 6.3 | 0.223 | 0.824 |
| Sludge, waste × Migration | 1.65 | 4.24 × 10^6^ | 3.9 × 10^-7^ | 1 |
| Soil × Migration | 1.62 | 3.35 × 10^6^ | 4.83 × 10^-7^ | 1 |

**Supplementary file 2l: Summary of the selected model for APHb for worldwide data.** *: p<0.05, **: p<0.01, ***: p<0.001.

| Autoregression coefficient φ = 2.17 × 10^-2^, Matérn smoothness ν = 16.7, Matérn scaling ρ = 0.724 | | | | |
| --- | --- | --- | --- | --- |
| **Variable** | **Estimate** | **Conditional standard error** | **t** | **p** |
| Intercept | -5.96 | 0.603 | -9.88 | 0 *** |
| Domestic animals | -31.3 | 3.91 × 10^6^ | -8.01 × 10^-6^ | 1 |
| Farms | -1.63 | 0.648 | -2.51 | 1.2 × 10^-2^ * |
| Flora, fauna | 0.809 | 0.701 | 1.15 | 0.248 |
| Human samples | -31.3 | 8.32 × 10^5^ | -3.76 × 10^-5^ | 1 |
| Human habitat | -9.06 × 10^-2^ | 0.902 | -0.1 | 0.92 |
| Freshwater | -31.2 | 2.34 × 10^6^ | -1.33 × 10^-5^ | 1 |
| Clinical samples | -4.82 | 1.15 | -4.19 | 2.74 × 10^-5^ *** |
| Sludge, waste | -31.1 | 3.57 × 10^6^ | -8.7 × 10^-6^ | 1 |
| Soil | 1.26 | 0.528 | 2.39 | 1.71 × 10^-2^ * |
| Trade | -0.221 | 0.229 | -0.968 | 0.333 |
| Migration | -0.24 | 0.18 | -1.34 | 0.181 |

**Supplementary file 2m: Summary of the selected model for APHd1 for worldwide data.** *: p<0.05, **: p<0.01, ***: p<0.001.

| Autoregression coefficient φ = 0.704, Matérn smoothness ν = 5.7 × 10^-3^, Matérn scaling ρ = 0.178 | | | | |
| --- | --- | --- | --- | --- |
| **Variable** | **Estimate** | **Conditional standard error** | **t** | **p** |
| Intercept | -36.9 | 3.13 × 10^6^ | -1.18 × 10^-5^ | 1 |
| Domestic animals | -7.7 | 5 × 10^6^ | -1.54 × 10^-6^ | 1 |
| Farms | -7.01 | 3.25 × 10^6^ | -2.16 × 10^-6^ | 1 |
| Flora, fauna | -7.1 | 4.95 × 10^6^ | -1.43 × 10^-6^ | 1 |
| Human samples | 25.1 | 3.13 × 10^6^ | 8.03 × 10^-6^ | 1 |
| Human habitat | 29.3 | 3.13 × 10^6^ | 9.37 × 10^-6^ | 1 |
| Freshwater | 27.9 | 3.13 × 10^6^ | 8.93 × 10^-6^ | 1 |
| Clinical samples | 27.7 | 3.13 × 10^6^ | 8.87 × 10^-6^ | 1 |
| Sludge, waste | 27.7 | 3.13 × 10^6^ | 8.87 × 10^-6^ | 1 |
| Soil | -6.69 | 3.68 × 10^6^ | -1.82 × 10^-6^ | 1 |
| Trade | 0.163 | 0.134 | 1.22 | 0.224 |
| Migration | -2.89 | 0.862 | -3.35 | 8.09 × 10^-4^ *** |

**Supplementary file 2n: Summary of the selected model for AACh for worldwide data.** *: p<0.05, **: p<0.01, ***: p<0.001.

| Autoregression coefficient φ = 0.931, Matérn smoothness ν = 1.88 × 10^-2^, Matérn scaling ρ = 1.89 | | | | |
| --- | --- | --- | --- | --- |
| **Variable** | **Estimate** | **Conditional standard error** | **t** | **p** |
| Intercept | -15.4 | 10.6 | -1.45 | 0.148 |
| Domestic animals | 8.65 | 10.6 | 0.816 | 0.414 |
| Farms | 9.64 | 10.6 | 0.912 | 0.362 |
| Flora, fauna | 10.6 | 10.6 | 1 | 0.316 |
| Human samples | 9.07 | 10.6 | 0.858 | 0.391 |
| Human habitat | 7.88 | 10.6 | 0.741 | 0.458 |
| Freshwater | -23.1 | 2.68 × 10^6^ | -8.61 × 10^-6^ | 1 |
| Clinical samples | 10.2 | 10.6 | 0.965 | 0.335 |
| Sludge, waste | 10.5 | 10.6 | 0.989 | 0.323 |
| Soil | 0.975 | 12.8 | 7.62 × 10^-2^ | 0.939 |
| Trade | 1.21 | 2.44 | 0.495 | 0.621 |
| Migration | 0.582 | 0.364 | 1.6 | 0.11 |
| Domestic animals × Trade | -0.813 | 2.47 | -0.329 | 0.742 |
| Farms × Trade | -1.61 | 2.45 | -0.66 | 0.509 |
| Flora, fauna × Trade | -1.68 | 2.47 | -0.679 | 0.497 |
| Human samples × Trade | -1.27 | 2.44 | -0.519 | 0.604 |
| Human habitat × Trade | -0.933 | 2.52 | -0.371 | 0.711 |
| Freshwater × Trade | -1.93 | 2.74 × 10^6^ | -7.06 × 10^-7^ | 1 |
| Clinical samples × Trade | -1.42 | 2.44 | -0.58 | 0.562 |
| Sludge, waste × Trade | -1.26 | 2.45 | -0.514 | 0.607 |
| Soil × Trade | -4 | 4.5 | -0.889 | 0.374 |
| Domestic animals × Migration | -0.717 | 0.417 | -1.72 | 8.57 × 10^-2^ * |
| Farms × Migration | -1.27 | 0.409 | -3.11 | 1.86 × 10^-3^ ** |
| Flora, fauna × Migration | -1.58 | 0.614 | -2.57 | 1.02 × 10^-2^ * |
| Human samples × Migration | -0.499 | 0.374 | -1.34 | 0.182 |
| Human habitat × Migration | -0.496 | 0.601 | -0.826 | 0.409 |
| Freshwater × Migration | -0.385 | 2.94 × 10^6^ | -1.31 × 10^-7^ | 1 |
| Clinical samples × Migration | -0.702 | 0.365 | -1.93 | 5.41 × 10^-2^ * |
| Sludge, waste × Migration | -0.682 | 0.411 | -1.66 | 9.68 × 10^-2^ * |
| Soil × Migration | -12.4 | 12.9 | -0.96 | 0.337 |

**Supplementary file 2o: Summary of the selected model for AACi for worldwide data.** *: p<0.05, **: p<0.01, ***: p<0.001.

| Autoregression coefficient φ = 0.283, Matérn smoothness ν = 16.7, Matérn scaling ρ = 0.823 | | | | |
| --- | --- | --- | --- | --- |
| **Variable** | **Estimate** | **Conditional standard error** | **t** | **p** |
| Intercept | -11 | 2.33 | -4.69 | 2.67 × 10^-6^ *** |
| Domestic animals | -26.9 | 4.38 × 10^6^ | -6.14 × 10^-6^ | 1 |
| Farms | 4.94 | 2.27 | 2.17 | 2.96 × 10^-2^ * |
| Flora, fauna | 2.49 | 4.18 | 0.595 | 0.552 |
| Human samples | 7.31 × 10^-2^ | 2.67 | 2.74 × 10^-2^ | 0.978 |
| Human habitat | -26.4 | 3.88 × 10^6^ | -6.8 × 10^-6^ | 1 |
| Freshwater | -26.9 | 2.53 × 10^6^ | -1.06 × 10^-5^ | 1 |
| Clinical samples | 3.34 | 2.28 | 1.46 | 0.144 |
| Sludge, waste | -27.1 | 5.52 × 10^6^ | -4.9 × 10^-6^ | 1 |
| Soil | -26.6 | 2.1 × 10^6^ | -1.27 × 10^-5^ | 1 |
| Trade | 0.289 | 0.493 | 0.586 | 0.558 |
| Migration | -10.8 | 7.07 | -1.52 | 0.128 |
| Domestic animals × Trade | -0.844 | 2.89 × 10^6^ | -2.92 × 10^-7^ | 1 |
| Farms × Trade | -0.413 | 0.492 | -0.84 | 0.401 |
| Flora, fauna × Trade | -1.65 | 3.5 | -0.471 | 0.638 |
| Human samples × Trade | -0.289 | 0.533 | -0.543 | 0.587 |
| Human habitat × Trade | -0.84 | 2.03 × 10^6^ | -4.14 × 10^-7^ | 1 |
| Freshwater × Trade | -0.772 | 1.45 × 10^6^ | -5.33 × 10^-7^ | 1 |
| Clinical samples × Trade | -1.03 | 0.531 | -1.93 | 5.31 × 10^-2^ * |
| Sludge, waste × Trade | -0.85 | 2.45 × 10^6^ | -3.47 × 10^-7^ | 1 |
| Soil × Trade | -1.06 | 2.08 × 10^6^ | -5.1 × 10^-7^ | 1 |
| Domestic animals × Migration | 10.6 | 2.74 × 10^6^ | 3.86 × 10^-6^ | 1 |
| Farms × Migration | 10.8 | 7.07 | 1.53 | 0.127 |
| Flora, fauna × Migration | 7.83 | 14.1 | 0.556 | 0.578 |
| Human samples × Migration | -1.99 | 8.09 | -0.246 | 0.806 |
| Human habitat × Migration | 10.2 | 5.3 × 10^6^ | 1.92 × 10^-6^ | 1 |
| Freshwater × Migration | 10.2 | 3.1 × 10^6^ | 3.28 × 10^-6^ | 1 |
| Clinical samples × Migration | 10.2 | 7.07 | 1.45 | 0.148 |
| Sludge, waste × Migration | 10.5 | 4.13 × 10^6^ | 2.55 × 10^-6^ | 1 |
| Soil × Migration | 10.5 | 3.27 × 10^6^ | 3.2 × 10^-6^ | 1 |

**Supplementary file 2p: Summary of the selected model for AACj for worldwide data.** *: p<0.05, **: p<0.01, ***: p<0.001.

| Autoregression coefficient φ = 0.521, Matérn smoothness ν = 1.22, Matérn scaling ρ = 0.534 | | | | |
| --- | --- | --- | --- | --- |
| **Variable** | **Estimate** | **Conditional standard error** | **t** | **p** |
| Intercept | -25.7 | 4.1 × 10^3^ | -6.27 × 10^-3^ | 0.995 |
| Domestic animals | -4.01 × 10^2^ | 4.8 × 10^6^ | -8.36 × 10^-5^ | 1 |
| Farms | -4.01 × 10^2^ | 1.09 × 10^6^ | -3.66 × 10^-4^ | 1 |
| Flora, fauna | -4 × 10^2^ | 4.13 × 10^6^ | -9.69 × 10^-5^ | 1 |
| Human samples | 18.6 | 4.1 × 10^3^ | 4.54 × 10^-3^ | 0.996 |
| Human habitat | -29.7 | 4.1 × 10^3^ | -7.23 × 10^-3^ | 0.994 |
| Freshwater | 17 | 4.1 × 10^3^ | 4.13 × 10^-3^ | 0.997 |
| Clinical samples | 19.8 | 4.1 × 10^3^ | 4.84 × 10^-3^ | 0.996 |
| Sludge, waste | -2.03 × 10^2^ | 6.25 × 10^4^ | -3.24 × 10^-3^ | 0.997 |
| Soil | 12.9 | 4.1 × 10^3^ | 3.13 × 10^-3^ | 0.997 |
| Trade | -0.717 | 3.22 × 10^3^ | -2.23 × 10^-4^ | 1 |
| Migration | 0.102 | 2.9 × 10^3^ | 3.53 × 10^-5^ | 1 |
| Domestic animals × Trade | 4.19 | 3.26 × 10^6^ | 1.29 × 10^-6^ | 1 |
| Farms × Trade | 2.46 | 5.65 × 10^5^ | 4.35 × 10^-6^ | 1 |
| Flora, fauna × Trade | 1.5 | 2.35 × 10^6^ | 6.38 × 10^-7^ | 1 |
| Human samples × Trade | 0.91 | 3.22 × 10^3^ | 2.83 × 10^-4^ | 1 |
| Human habitat × Trade | 2.83 | 3.22 × 10^3^ | 8.79 × 10^-4^ | 0.999 |
| Freshwater × Trade | -0.351 | 3.22 × 10^3^ | -1.09 × 10^-4^ | 1 |
| Clinical samples × Trade | 0.423 | 3.22 × 10^3^ | 1.31 × 10^-4^ | 1 |
| Sludge, waste × Trade | -2.58 × 10^2^ | 1.57 × 10^5^ | -1.64 × 10^-3^ | 0.999 |
| Soil × Trade | -6.99 | 3.22 × 10^3^ | -2.17 × 10^-3^ | 0.998 |
| Domestic animals × Migration | -0.821 | 2.08 × 10^6^ | -3.95 × 10^-7^ | 1 |
| Farms × Migration | -0.64 | 3.4 × 10^5^ | -1.88 × 10^-6^ | 1 |
| Flora, fauna × Migration | 6.75 × 10^-2^ | 2.53 × 10^6^ | 2.67 × 10^-8^ | 1 |
| Human samples × Migration | -2.87 | 2.9 × 10^3^ | -9.9 × 10^-4^ | 0.999 |
| Human habitat × Migration | -1.63 × 10^2^ | 2.91 × 10^3^ | -5.62 × 10^-2^ | 0.955 |
| Freshwater × Migration | -1.54 | 2.9 × 10^3^ | -5.29 × 10^-4^ | 1 |
| Clinical samples × Migration | -0.195 | 2.9 × 10^3^ | -6.71 × 10^-5^ | 1 |
| Sludge, waste × Migration | -1.94 × 10^2^ | 3.11 × 10^5^ | -6.24 × 10^-4^ | 1 |
| Soil × Migration | -5.92 | 2.9 × 10^3^ | -2.04 × 10^-3^ | 0.998 |

**Supplementary file 2q: Summary of the selected model for ANTe for worldwide data.** *: p<0.05, **: p<0.01, ***: p<0.001.

| Autoregression coefficient φ = -0.469, Matérn smoothness ν = 5.01 × 10^-3^, Matérn scaling ρ = 2.54 × 10^-2^ | | | | |
| --- | --- | --- | --- | --- |
| **Variable** | **Estimate** | **Conditional standard error** | **t** | **p** |
| Intercept | -39.6 | 25.7 | -1.54 | 0.123 |
| Trade | -28.9 | 36.1 | -0.801 | 0.423 |
| Migration | -1.23 × 10^2^ | 1.43 × 10^2^ | -0.856 | 0.392 |

**Supplementary file 2r: Summary of the selected model for APHf for worldwide data.** *: p<0.05, **: p<0.01, ***: p<0.001.

| Autoregression coefficient φ = 1.14 × 10^-2^, Matérn smoothness ν = 8.21 × 10^-2^, Matérn scaling ρ = 1.29 × 10^-2^ | | | | |
| --- | --- | --- | --- | --- |
| **Variable** | **Estimate** | **Conditional standard error** | **t** | **p** |
| Intercept | -4.61 | 0.549 | -8.39 | 0 *** |
| Domestic animals | -32.5 | 4.54 × 10^6^ | -7.15 × 10^-6^ | 1 |
| Farms | -2.1 | 0.509 | -4.12 | 3.76 × 10^-5^ *** |
| Flora, fauna | -0.698 | 0.85 | -0.821 | 0.412 |
| Human samples | -1.45 | 0.317 | -4.58 | 4.66 × 10^-6^ *** |
| Human habitat | -3.63 | 3.63 | -0.999 | 0.318 |
| Freshwater | -0.198 | 0.367 | -0.541 | 0.589 |
| Clinical samples | -0.395 | 0.209 | -1.89 | 5.84 × 10^-2^ * |
| Sludge, waste | -0.156 | 0.659 | -0.236 | 0.813 |
| Soil | -0.437 | 0.389 | -1.12 | 0.261 |
| Trade | -5.93 × 10^-2^ | 8.65 × 10^-2^ | -0.686 | 0.493 |
| Migration | 0.651 | 8.8 × 10^-2^ | 7.39 | 1.45 × 10^-13^ *** |
| Domestic animals × Trade | -0.464 | 3.5 × 10^6^ | -1.33 × 10^-7^ | 1 |
| Farms × Trade | 6.4 × 10^-2^ | 0.119 | 0.539 | 0.59 |
| Flora, fauna × Trade | -0.198 | 0.447 | -0.444 | 0.657 |
| Human samples × Trade | -0.483 | 0.268 | -1.8 | 7.16 × 10^-2^ * |
| Human habitat × Trade | 2.75 × 10^-2^ | 0.319 | 8.63 × 10^-2^ | 0.931 |
| Freshwater × Trade | -1.38 | 0.716 | -1.92 | 5.47 × 10^-2^ * |
| Clinical samples × Trade | -1.09 | 0.175 | -6.26 | 3.79 × 10^-10^ *** |
| Sludge, waste × Trade | -0.256 | 0.284 | -0.901 | 0.368 |
| Soil × Trade | -0.323 | 0.322 | -1 | 0.316 |
| Domestic animals × Migration | -0.421 | 3.61 × 10^6^ | -1.17 × 10^-7^ | 1 |
| Farms × Migration | -2.99 | 1.08 | -2.76 | 5.82 × 10^-3^ ** |
| Flora, fauna × Migration | -0.776 | 0.7 | -1.11 | 0.268 |
| Human samples × Migration | -1.02 | 0.312 | -3.27 | 1.08 × 10^-3^ ** |
| Human habitat × Migration | -10.2 | 8.7 | -1.17 | 0.243 |
| Freshwater × Migration | -1.05 | 0.426 | -2.47 | 1.33 × 10^-2^ * |
| Clinical samples × Migration | -0.883 | 0.148 | -5.95 | 2.65 × 10^-9^ *** |
| Sludge, waste × Migration | -0.665 | 0.477 | -1.4 | 0.163 |
| Soil × Migration | -7.66 × 10^-3^ | 0.306 | -2.51 × 10^-2^ | 0.98 |
