# Supplementary material for: Ecology, more than antibiotics consumption, is the major predictor for the global distribution of aminoglycoside-modifying enzymes
Source: eLife. 2023 Feb 14;12:e77015. doi: 10.7554/eLife.77015 (PMC9928423; doi:10.7554/eLife.77015)
Supplement: Supplementary file 3. [file elife-77015-supp3.doc]

**Supplementary file 3a: Summary of the selected model for AACc under low sampling.** *: p<0.05, **: p<0.01, ***: p<0.001.

| Autoregression coefficient φ = -0.9, Matérn smoothness ν = 5.0 × 10^-3^, Matérn scaling ρ = 6.1 | | | | |
| --- | --- | --- | --- | --- |
| **Variable** | **Estimate** | **Conditional standard error** | **t** | **p** |
| *Intercept* | -13.1 | 4.0 | -3.3 | 1.1 × 10^-3^ ** |
| *Domestic animals* | -2.4 × 10^2^ | 8.6 × 10^6^ | -2.8 × 10^-5^ | 1.0 |
| *Farms* | -4.5 × 10^2^ | 2.1 × 10^6^ | -2.1 × 10^-4^ | 1.0 |
| *Flora, fauna* | -4.6 × 10^2^ | 1.9 × 10^7^ | -2.4 × 10^-5^ | 1.0 |
| *Human samples* | -0.6 | 1.3 | -0.5 | 0.6 |
| *Human habitat* | -3.8 × 10^2^ | 1.9 × 10^7^ | -2.0 × 10^-5^ | 1.0 |
| *Freshwater* | -1.1 × 10^3^ | 1.2 × 10^7^ | -9.0 × 10^-5^ | 1.0 |
| *Clinical samples* | 4.2 × 10^-2^ | 1.1 | 3.8 × 10^-2^ | 1.0 |
| *Sludge, waste* | -6.2 × 10^2^ | 6.8 × 10^6^ | -9.1 × 10^-5^ | 1.0 |
| *Soil* | -0.4 | 1.6 | -0.2 | 0.8 |
| *Aminoglycosides* | 1.2 | 2.6 | 0.5 | 0.6 |
| *Trade* | 0.9 | 0.4 | 2.0 | 4.0 × 10^-2^ * |
| *Migration* | -0.2 | 0.4 | -0.5 | 0.6 |
| *Domestic animals × Aminoglycosides* | 56.5 | 1.6 × 10^7^ | 3.5 × 10^-6^ | 1.0 |
| *Farms × Aminoglycosides* | 76.9 | 4.3 × 10^5^ | 1.8 × 10^-4^ | 1.0 |
| *Flora, fauna × Aminoglycosides* | 4.1 × 10^2^ | 4.7 × 10^7^ | 8.8 × 10^-6^ | 1.0 |
| *Human samples × Aminoglycosides* | -1.3 | 2.9 | -0.5 | 0.6 |
| *Human habitat × Aminoglycosides* | 2.0 × 10^2^ | 3.2 × 10^7^ | 6.5 × 10^-6^ | 1.0 |
| *Freshwater × Aminoglycosides* | 6.1 × 10^2^ | 1.5 × 10^7^ | 4.0 × 10^-5^ | 1.0 |
| *Clinical samples × Aminoglycosides* | 2.1 | 2.6 | 0.8 | 0.4 |
| *Sludge, waste × Aminoglycosides* | 4.8 × 10^2^ | 5.5 × 10^6^ | 8.8 × 10^-5^ | 1.0 |
| *Soil × Aminoglycosides* | -2.2 | 3.6 | -0.6 | 0.5 |

**Supplementary file 3b: Summary of the selected model for AACc under high sampling.** *: p<0.05, **: p<0.01, ***: p<0.001.

| Autoregression coefficient φ = 0.7, Matérn smoothness ν = 5.0, Matérn scaling ρ = 0.2 | | | | |
| --- | --- | --- | --- | --- |
| **Variable** | **Estimate** | **Conditional standard error** | **t** | **p** |
| *Intercept* | -45.0 | 2.3 × 10^3^ | -2.0 × 10^-2^ | 1.0 |
| *Domestic animals* | -4.7 × 10^2^ | 8.1 × 10^6^ | -5.8 × 10^-5^ | 1.0 |
| *Farms* | -1.6 × 10^3^ | 2.1 × 10^6^ | -7.8 × 10^-4^ | 1.0 |
| *Flora, fauna* | -5.8 × 10^2^ | 1.1 × 10^7^ | -5.5 × 10^-5^ | 1.0 |
| *Human samples* | -6.4 × 10^3^ | 2.7 × 10^6^ | -2.4 × 10^-3^ | 1.0 |
| *Human habitat* | -1.0 × 10^4^ | 1.5 × 10^7^ | -7.1 × 10^-4^ | 1.0 |
| *Freshwater* | -1.6 × 10^4^ | 1.2 × 10^7^ | -1.4 × 10^-3^ | 1.0 |
| *Clinical samples* | 23.5 | 2.3 × 10^3^ | 1.0 × 10^-2^ | 1.0 |
| *Sludge, waste* | -3.4 × 10^2^ | 5.4 × 10^6^ | -6.3 × 10^-5^ | 1.0 |
| *Soil* | -5.2 × 10^3^ | 7.9 × 10^6^ | -6.6 × 10^-4^ | 1.0 |
| *Trade* | -1.4 | 1.1 | -1.2 | 0.2 |
| *Migration* | 19.0 | 2.7 | 7.0 | 2.8 × 10^-12^ *** |

**Supplementary file 3c: Summary of the selected model for AACe1 under low sampling.** *: p<0.05, **: p<0.01, ***: p<0.001.

| Autoregression coefficient φ = 5.5 × 10^-7^, Matérn smoothness ν = 16.7, Matérn scaling ρ = 2.3 | | | | |
| --- | --- | --- | --- | --- |
| **Variable** | **Estimate** | **Conditional standard error** | **t** | **p** |
| *Intercept* | -5.4 | 0.9 | -5.9 | 2.9 × 10^-9^ *** |
| *Domestic animals* | -31.8 | 8.5 × 10^6^ | -3.8 × 10^-6^ | 1.0 |
| *Farms* | -31.4 | 2.3 × 10^6^ | -1.4 × 10^-5^ | 1.0 |
| *Flora, fauna* | 2.4 | 1.1 | 2.3 | 2.0 × 10^-2^ * |
| *Human samples* | 2.1 | 0.8 | 2.8 | 5.1 × 10^-3^ ** |
| *Human habitat* | 2.5 | 1.3 | 1.9 | 5.4 × 10^-2^ |
| *Freshwater* | -31.5 | 1.3 × 10^7^ | -2.5 × 10^-6^ | 1.0 |
| *Clinical samples* | 1.9 | 0.7 | 2.6 | 8.6 × 10^-3^ ** |
| *Sludge, waste* | 3.5 | 0.9 | 4.0 | 5.2 × 10^-5^ *** |
| *Soil* | -31.6 | 8.4 × 10^6^ | -3.8 × 10^-6^ | 1.0 |

**Supplementary file 3d: Summary of the selected model for AACe1 under high sampling.** *: p<0.05, **: p<0.01, ***: p<0.001.

| Autoregression coefficient φ = 0.8, Matérn smoothness ν = 0.2, Matérn scaling ρ = 5.5 × 10^-2^ | | | | |
| --- | --- | --- | --- | --- |
| **Variable** | **Estimate** | **Conditional standard error** | **t** | **p** |
| *Intercept* | -20.7 | 2.4 × 10^3^ | -8.7 × 10^-3^ | 1.0 |
| *Domestic animals* | -1.2 × 10^2^ | 1.2 × 10^7^ | -9.2 × 10^-6^ | 1.0 |
| *Farms* | 10.9 | 2.4 × 10^3^ | 4.5 × 10^-3^ | 1.0 |
| *Flora, fauna* | -2.9 × 10^2^ | 1.2 × 10^8^ | -2.4 × 10^-6^ | 1.0 |
| *Human samples* | -1.0 × 10^2^ | 3.6 × 10^6^ | -2.8 × 10^-5^ | 1.0 |
| *Human habitat* | 21.1 | 1.4 × 10^7^ | 1.5 × 10^-6^ | 1.0 |
| *Freshwater* | -1.6 × 10^2^ | 1.2 × 10^7^ | -1.3 × 10^-5^ | 1.0 |
| *Clinical samples* | 18.4 | 2.4 × 10^3^ | 7.7 × 10^-3^ | 1.0 |
| *Sludge, waste* | 88.6 | 8.4 × 10^7^ | 1.1 × 10^-6^ | 1.0 |
| *Soil* | -1.2 × 10^2^ | 1.1 × 10^7^ | -1.1 × 10^-5^ | 1.0 |
| *Trade* | -0.2 | 2.4 × 10^3^ | -8.7 × 10^-5^ | 1.0 |
| *Migration* | -0.7 | 3.2 × 10^3^ | -2.3 × 10^-4^ | 1.0 |
| *Domestic animals × Trade* | 97.9 | 2.1 × 10^7^ | 4.6 × 10^-6^ | 1.0 |
| *Farms × Trade* | 6.3 | 2.4 × 10^3^ | 2.6 × 10^-3^ | 1.0 |
| *Flora, fauna × Trade* | -25.0 | 1.0 × 10^8^ | -2.4 × 10^-7^ | 1.0 |
| *Human samples × Trade* | -3.3 | 7.8 × 10^6^ | -4.2 × 10^-7^ | 1.0 |
| *Human habitat × Trade* | -1.0 | 1.8 × 10^7^ | -5.5 × 10^-8^ | 1.0 |
| *Freshwater × Trade* | -33.0 | 1.7 × 10^7^ | -1.9 × 10^-6^ | 1.0 |
| *Clinical samples × Trade* | 0.5 | 2.4 × 10^3^ | 2.2 × 10^-4^ | 1.0 |
| *Sludge, waste × Trade* | -2.4 × 10^2^ | 4.5 × 10^7^ | -5.3 × 10^-6^ | 1.0 |
| *Soil × Trade* | -9.9 | 1.8 × 10^7^ | -5.6 × 10^-7^ | 1.0 |
| *Domestic animals × Migration* | -49.6 | 2.7 × 10^7^ | -1.8 × 10^-6^ | 1.0 |
| *Farms × Migration* | -3.6 | 3.2 × 10^3^ | -1.1 × 10^-3^ | 1.0 |
| *Flora, fauna × Migration* | 77.7 | 2.5 × 10^8^ | 3.1 × 10^-7^ | 1.0 |
| *Human samples × Migration* | 7.6 | 9.0 × 10^6^ | 8.5 × 10^-7^ | 1.0 |
| *Human habitat × Migration* | -51.7 | 1.9 × 10^7^ | -2.7 × 10^-6^ | 1.0 |
| *Freshwater × Migration* | -6.5 | 1.5 × 10^7^ | -4.2 × 10^-7^ | 1.0 |
| *Clinical samples × Migration* | 0.5 | 3.2 × 10^3^ | 1.4 × 10^-4^ | 1.0 |
| *Sludge, waste × Migration* | NA | NA | NA | NA |
| *Soil × Migration* | 11.8 | 1.6 × 10^7^ | 7.2 × 10^-7^ | 1.0 |

**Supplementary file 3e: Summary of the selected model for AACf1 under low sampling.** *: p<0.05, **: p<0.01, ***: p<0.001.

| Autoregression coefficient φ = 0.9, Matérn smoothness ν = 1.1, Matérn scaling ρ = 0.3 | | | | |
| --- | --- | --- | --- | --- |
| **Variable** | **Estimate** | **Conditional standard error** | **t** | **p** |
| *Intercept* | -4.4 | 1.3 | -3.4 | 6.0 × 10^-4^ *** |
| *Domestic animals* | -2.0 × 10^2^ | 2.1 × 10^6^ | -9.2 × 10^-5^ | 1.0 |
| *Farms* | 0.4 | 0.8 | 0.5 | 0.6 |
| *Flora, fauna* | -33.8 | 1.6 × 10^7^ | -2.0 × 10^-6^ | 1.0 |
| *Human samples* | -3.7 | 1.9 | -2.0 | 4.9 × 10^-2^ * |
| *Human habitat* | 2.0 | 1.1 | 1.8 | 7.6 × 10^-2^ |
| *Freshwater* | -2.6 | 3.1 | -0.8 | 0.4 |
| *Clinical samples* | -1.1 | 0.8 | -1.4 | 0.2 |
| *Sludge, waste* | -1.9 × 10^2^ | 1.6 × 10^6^ | -1.2 × 10^-4^ | 1.0 |
| *Soil* | 1.5 | 0.9 | 1.7 | 9.3 × 10^-2^ |
| *Aminoglycosides* | -1.6 | 1.4 | -1.1 | 0.3 |
| *Trade* | -0.3 | 0.7 | -0.4 | 0.7 |
| *Migration* | -0.8 | 0.6 | -1.4 | 0.2 |
| *Domestic animals × Aminoglycosides* | -1.4 × 10^2^ | 1.2 × 10^7^ | -1.1 × 10^-5^ | 1.0 |
| *Farms × Aminoglycosides* | 0.7 | 1.4 | 0.5 | 0.6 |
| *Flora, fauna × Aminoglycosides* | 0.2 | 3.6 × 10^7^ | 6.3 × 10^-9^ | 1.0 |
| *Human samples × Aminoglycosides* | 1.5 | 3.3 | 0.5 | 0.6 |
| *Human habitat × Aminoglycosides* | 1.6 | 2.0 | 0.8 | 0.4 |
| *Freshwater × Aminoglycosides* | -4.8 | 4.5 | -1.1 | 0.3 |
| *Clinical samples × Aminoglycosides* | 2.1 | 1.4 | 1.5 | 0.1 |
| *Sludge, waste × Aminoglycosides* | -2.4 × 10^2^ | 2.2 × 10^6^ | -1.1 × 10^-4^ | 1.0 |
| *Soil × Aminoglycosides* | 3.8 | 1.6 | 2.4 | 1.5 × 10^-2^ * |
| *Domestic animals × Trade* | 37.9 | 6.7 × 10^5^ | 5.7 × 10^-5^ | 1.0 |
| *Farms × Trade* | -0.6 | 0.7 | -0.9 | 0.4 |
| *Flora, fauna × Trade* | 1.1 | 1.4 × 10^7^ | 7.9 × 10^-8^ | 1.0 |
| *Human samples × Trade* | 0.2 | 1.7 | 0.1 | 0.9 |
| *Human habitat × Trade* | 1.2 | 1.2 | 1.0 | 0.3 |
| *Freshwater × Trade* | 3.4 | 1.5 | 2.3 | 2.3 × 10^-2^ * |
| *Clinical samples × Trade* | 7.1 × 10^-3^ | 0.7 | 1.1 × 10^-2^ | 1.0 |
| *Sludge, waste × Trade* | -1.3 × 10^2^ | 1.1 × 10^6^ | -1.2 × 10^-4^ | 1.0 |
| *Soil × Trade* | -1.3 | 1.0 | -1.4 | 0.2 |
| *Domestic animals × Migration* | 62.7 | 5.0 × 10^6^ | 1.3 × 10^-5^ | 1.0 |
| *Farms × Migration* | 1.6 | 0.5 | 3.0 | 2.6 × 10^-3^ ** |
| *Flora, fauna × Migration* | 0.6 | 1.3 × 10^7^ | 4.2 × 10^-8^ | 1.0 |
| *Human samples × Migration* | 0.1 | 2.3 | 4.9 × 10^-2^ | 1.0 |
| *Human habitat × Migration* | -0.7 | 1.6 | -0.5 | 0.6 |
| *Freshwater × Migration* | -2.2 | 1.6 | -1.4 | 0.2 |
| *Clinical samples × Migration* | 1.1 | 0.6 | 1.9 | 6.3 × 10^-2^ |
| *Sludge, waste × Migration* | -23.9 | 2.9 × 10^5^ | -8.2 × 10^-5^ | 1.0 |
| *Soil × Migration* | 1.0 | 0.9 | 1.1 | 0.3 |

**Supplementary file 3f: Summary of the selected model for AACf1 under high sampling.** *: p<0.05, **: p<0.01, ***: p<0.001.

| Autoregression coefficient φ = 0.6, Matérn smoothness ν = 9.2 × 10^-2^, Matérn scaling ρ = 2.6 | | | | |
| --- | --- | --- | --- | --- |
| **Variable** | **Estimate** | **Conditional standard error** | **t** | **p** |
| *Intercept* | -11.3 | 2.3 | -4.8 | 1.3 × 10^-6^ *** |
| *Domestic animals* | -64.9 | 2.4 × 10^7^ | -2.8 × 10^-6^ | 1.0 |
| *Farms* | -1.1 | 1.7 | -0.7 | 0.5 |
| *Flora, fauna* | -55.2 | 2.0 × 10^7^ | -2.7 × 10^-6^ | 1.0 |
| *Human samples* | 8.1 | 1.7 | 4.8 | 1.8 × 10^-6^ *** |
| *Human habitat* | -16.2 | 1.2 × 10^8^ | -1.4 × 10^-7^ | 1.0 |
| *Freshwater* | -1.1 × 10^3^ | 1.0 × 10^7^ | -1.1 × 10^-4^ | 1.0 |
| *Clinical samples* | -1.3 × 10^2^ | 1.2 × 10^6^ | -1.1 × 10^-4^ | 1.0 |
| *Sludge, waste* | -1.7 × 10^2^ | 7.0 × 10^6^ | -2.5 × 10^-5^ | 1.0 |
| *Soil* | 2.0 | 2.1 | 0.9 | 0.3 |
| *Aminoglycosides* | -2.9 | 0.7 | -4.0 | 5.9 × 10^-5^ *** |
| *Trade* | 4.1 | 1.8 | 2.3 | 2.2 × 10^-2^ * |
| *Migration* | -2.1 | 1.2 | -1.7 | 9.1 × 10^-2^ |
| *Domestic animals × Trade* | 6.0 | 3.8 × 10^7^ | 1.6 × 10^-7^ | 1.0 |
| *Farms × Trade* | -3.7 | 2.2 | -1.6 | 10.0 × 10^-2^ |
| *Flora, fauna × Trade* | -29.2 | 3.0 × 10^8^ | -9.6 × 10^-8^ | 1.0 |
| *Human samples × Trade* | -7.1 | 1.9 | -3.7 | 2.3 × 10^-4^ *** |
| *Human habitat × Trade* | -37.9 | 8.0 × 10^7^ | -4.7 × 10^-7^ | 1.0 |
| *Freshwater × Trade* | -9.7 × 10^2^ | 1.5 × 10^7^ | -6.4 × 10^-5^ | 1.0 |
| *Clinical samples × Trade* | 46.6 | 1.5 × 10^6^ | 3.1 × 10^-5^ | 1.0 |
| *Sludge, waste × Trade* | 14.3 | 1.9 × 10^7^ | 7.7 × 10^-7^ | 1.0 |
| *Soil × Trade* | -9.1 | 4.1 | -2.2 | 2.8 × 10^-2^ * |
| *Domestic animals × Migration* | -14.7 | 3.8 × 10^7^ | -3.9 × 10^-7^ | 1.0 |
| *Farms × Migration* | 3.7 | 1.9 | 1.9 | 5.7 × 10^-2^ |
| *Flora, fauna × Migration* | 7.8 | 4.8 × 10^7^ | 1.6 × 10^-7^ | 1.0 |
| *Human samples × Migration* | 2.6 | 1.3 | 2.0 | 4.9 × 10^-2^ * |
| *Human habitat × Migration* | 73.1 | 1.8 × 10^8^ | 4.0 × 10^-7^ | 1.0 |
| *Freshwater × Migration* | -13.0 | 9.2 × 10^6^ | -1.4 × 10^-6^ | 1.0 |
| *Clinical samples × Migration* | 1.4 | 1.8 × 10^6^ | 7.5 × 10^-7^ | 1.0 |
| *Sludge, waste × Migration* | NA | NA | NA | NA |
| *Soil × Migration* | -1.9 | 6.8 | -0.3 | 0.8 |

**Supplementary file 3g: Summary of the selected model for AACg under low sampling.** *: p<0.05, **: p<0.01, ***: p<0.001.

| Autoregression coefficient φ = -4.7 × 10^-7^, Matérn smoothness ν = 8.2 × 10^-2^, Matérn scaling ρ = 6.6 | | | | |
| --- | --- | --- | --- | --- |
| **Variable** | **Estimate** | **Conditional standard error** | **t** | **p** |
| *Intercept* | -16.5 | 6.8 | -2.4 | 1.6 × 10^-2^ * |
| *Domestic animals* | -0.8 | 21.7 | -3.5 × 10^-2^ | 1.0 |
| *Farms* | 12.1 | 6.8 | 1.8 | 7.6 × 10^-2^ |
| *Flora, fauna* | -1.1 × 10^3^ | 1.7 × 10^7^ | -6.5 × 10^-5^ | 1.0 |
| *Human samples* | 10.3 | 6.9 | 1.5 | 0.1 |
| *Human habitat* | -51.8 | 1.6 × 10^5^ | -3.2 × 10^-4^ | 1.0 |
| *Freshwater* | -3.3 × 10^2^ | 1.2 × 10^7^ | -2.7 × 10^-5^ | 1.0 |
| *Clinical samples* | 10.3 | 6.8 | 1.5 | 0.1 |
| *Sludge, waste* | -3.3 × 10^2^ | 1.5 × 10^7^ | -2.3 × 10^-5^ | 1.0 |
| *Soil* | -7.9 × 10^2^ | 8.9 × 10^6^ | -8.9 × 10^-5^ | 1.0 |
| *Aminoglycosides* | -13.1 | 4.0 | -3.2 | 1.2 × 10^-3^ ** |
| *Trade* | 4.3 | 2.6 | 1.6 | 10.0 × 10^-2^ |
| *Migration* | 0.3 | 1.2 | 0.3 | 0.8 |
| *Domestic animals × Aminoglycosides* | 19.3 | 9.5 | 2.0 | 4.3 × 10^-2^ * |
| *Farms × Aminoglycosides* | 12.9 | 4.0 | 3.2 | 1.4 × 10^-3^ ** |
| *Flora, fauna × Aminoglycosides* | -4.9 × 10^2^ | 4.0 × 10^7^ | -1.2 × 10^-5^ | 1.0 |
| *Human samples × Aminoglycosides* | 11.9 | 4.4 | 2.7 | 6.4 × 10^-3^ ** |
| *Human habitat × Aminoglycosides* | -33.6 | 2.2 × 10^5^ | -1.5 × 10^-4^ | 1.0 |
| *Freshwater × Aminoglycosides* | 17.0 | 1.7 × 10^7^ | 1.0 × 10^-6^ | 1.0 |
| *Clinical samples × Aminoglycosides* | 12.3 | 4.0 | 3.1 | 2.2 × 10^-3^ ** |
| *Sludge, waste × Aminoglycosides* | 4.1 | 3.5 × 10^7^ | 1.2 × 10^-7^ | 1.0 |
| *Soil × Aminoglycosides* | -5.4 × 10^2^ | 1.3 × 10^7^ | -4.1 × 10^-5^ | 1.0 |
| *Domestic animals × Trade* | 0.4 | 7.5 | 4.8 × 10^-2^ | 1.0 |
| *Farms × Trade* | -3.9 | 2.6 | -1.5 | 0.1 |
| *Flora, fauna × Trade* | 1.5 × 10^2^ | 1.1 × 10^7^ | 1.5 × 10^-5^ | 1.0 |
| *Human samples × Trade* | -3.9 | 2.7 | -1.5 | 0.1 |
| *Human habitat × Trade* | 25.9 | 2.6 × 10^5^ | 1.0 × 10^-4^ | 1.0 |
| *Freshwater × Trade* | 26.2 | 1.4 × 10^7^ | 1.9 × 10^-6^ | 1.0 |
| *Clinical samples × Trade* | -3.6 | 2.6 | -1.4 | 0.2 |
| *Sludge, waste × Trade* | 24.9 | 1.2 × 10^7^ | 2.1 × 10^-6^ | 1.0 |
| *Soil × Trade* | 2.6 × 10^2^ | 3.4 × 10^6^ | 7.5 × 10^-5^ | 1.0 |
| *Domestic animals × Migration* | 3.0 | 5.3 | 0.6 | 0.6 |
| *Farms × Migration* | -0.7 | 1.2 | -0.6 | 0.5 |
| *Flora, fauna × Migration* | -9.8 × 10^2^ | 1.5 × 10^7^ | -6.6 × 10^-5^ | 1.0 |
| *Human samples × Migration* | 0.7 | 1.3 | 0.6 | 0.6 |
| *Human habitat × Migration* | -1.0 | 2.7 × 10^5^ | -3.5 × 10^-6^ | 1.0 |
| *Freshwater × Migration* | 9.4 | 1.4 × 10^7^ | 6.5 × 10^-7^ | 1.0 |
| *Clinical samples × Migration* | 1.4 | 1.2 | 1.2 | 0.2 |
| *Sludge, waste × Migration* | 20.9 | 1.4 × 10^7^ | 1.5 × 10^-6^ | 1.0 |
| *Soil × Migration* | -9.6 | 7.6 × 10^6^ | -1.2 × 10^-6^ | 1.0 |

**Supplementary file 3h: Summary of the selected model for AACg under high sampling.** *: p<0.05, **: p<0.01, ***: p<0.001.

| Autoregression coefficient φ = 0.6, Matérn smoothness ν = 7.1 × 10^-2^, Matérn scaling ρ = 7.1 | | | | |
| --- | --- | --- | --- | --- |
| **Variable** | **Estimate** | **Conditional standard error** | **t** | **p** |
| *Intercept* | -4.1 | 0.9 | -4.6 | 4.1 × 10^-6^ *** |
| *Domestic animals* | -0.8 | 1.7 | -0.5 | 0.6 |
| *Farms* | -4.3 × 10^2^ | 2.4 × 10^6^ | -1.8 × 10^-4^ | 1.0 |
| *Flora, fauna* | -5.3 × 10^2^ | 3.3 × 10^7^ | -1.6 × 10^-5^ | 1.0 |
| *Human samples* | 0.9 | 0.9 | 1.0 | 0.3 |
| *Human habitat* | -4.3 × 10^2^ | 1.4 × 10^5^ | -3.2 × 10^-3^ | 1.0 |
| *Freshwater* | -4.8 × 10^2^ | 1.2 × 10^7^ | -4.0 × 10^-5^ | 1.0 |
| *Clinical samples* | -0.4 | 0.9 | -0.5 | 0.6 |
| *Sludge, waste* | -6.4 × 10^2^ | 1.2 × 10^7^ | -5.3 × 10^-5^ | 1.0 |
| *Soil* | -4.8 × 10^2^ | 1.1 × 10^7^ | -4.2 × 10^-5^ | 1.0 |
| *Trade* | 0.2 | 0.5 | 0.3 | 0.8 |
| *Migration* | 1.1 | 1.0 | 1.0 | 0.3 |
| *Domestic animals × Trade* | 1.5 | 2.1 | 0.7 | 0.5 |
| *Farms × Trade* | 0.6 | 2.7 × 10^6^ | 2.3 × 10^-7^ | 1.0 |
| *Flora, fauna × Trade* | 2.5 × 10^2^ | 8.3 × 10^7^ | 3.0 × 10^-6^ | 1.0 |
| *Human samples × Trade* | 0.5 | 0.5 | 1.0 | 0.3 |
| *Human habitat × Trade* | 16.3 | 4.8 × 10^3^ | 3.4 × 10^-3^ | 1.0 |
| *Freshwater × Trade* | 10.9 | 9.9 × 10^6^ | 1.1 × 10^-6^ | 1.0 |
| *Clinical samples × Trade* | -0.8 | 0.5 | -1.4 | 0.2 |
| *Sludge, waste × Trade* | 48.3 | 1.4 × 10^7^ | 3.5 × 10^-6^ | 1.0 |
| *Soil × Trade* | -18.4 | 7.7 × 10^6^ | -2.4 × 10^-6^ | 1.0 |
| *Domestic animals × Migration* | -1.1 | 2.4 | -0.5 | 0.6 |
| *Farms × Migration* | 4.4 | 2.1 × 10^6^ | 2.1 × 10^-6^ | 1.0 |
| *Flora, fauna × Migration* | -46.5 | 2.8 × 10^7^ | -1.7 × 10^-6^ | 1.0 |
| *Human samples × Migration* | -0.9 | 1.1 | -0.8 | 0.4 |
| *Human habitat × Migration* | 3.1 × 10^2^ | 9.8 × 10^4^ | 3.2 × 10^-3^ | 1.0 |
| *Freshwater × Migration* | -19.8 | 1.2 × 10^7^ | -1.6 × 10^-6^ | 1.0 |
| *Clinical samples × Migration* | -1.0 | 1.0 | -1.0 | 0.3 |
| *Sludge, waste × Migration* | NA | NA | NA | NA |
| *Soil × Migration* | 24.0 | 7.8 × 10^6^ | 3.1 × 10^-6^ | 1.0 |

**Supplementary file 3i: Summary of the selected model for ANTa under low sampling.** *: p<0.05, **: p<0.01, ***: p<0.001.

| Autoregression coefficient φ = -4.5 × 10^-8^, Matérn smoothness ν = 0.2, Matérn scaling ρ = 11.2 | | | | |
| --- | --- | --- | --- | --- |
| **Variable** | **Estimate** | **Conditional standard error** | **t** | **p** |
| *Intercept* | -4.0 | 0.7 | -5.6 | 2.6 × 10^-8^ *** |
| *Domestic animals* | -31.2 | 24.8 | -1.3 | 0.2 |
| *Farms* | -0.6 | 0.8 | -0.8 | 0.4 |
| *Flora, fauna* | -4.5 | 6.2 | -0.7 | 0.5 |
| *Human samples* | -1.3 | 1.0 | -1.4 | 0.2 |
| *Human habitat* | -2.2 × 10^2^ | 4.8 × 10^6^ | -4.5 × 10^-5^ | 1.0 |
| *Freshwater* | -33.2 | 1.2 × 10^7^ | -2.7 × 10^-6^ | 1.0 |
| *Clinical samples* | -0.9 | 0.7 | -1.3 | 0.2 |
| *Sludge, waste* | -33.8 | 1.2 × 10^7^ | -2.9 × 10^-6^ | 1.0 |
| *Soil* | 0.4 | 1.0 | 0.4 | 0.7 |
| *Aminoglycosides* | 3.2 × 10^-2^ | 0.8 | 3.8 × 10^-2^ | 1.0 |
| *Trade* | -0.3 | 1.1 | -0.3 | 0.8 |
| *Migration* | -0.5 | 1.1 | -0.5 | 0.6 |
| *Domestic animals × Aminoglycosides* | 4.8 | 3.8 | 1.3 | 0.2 |
| *Farms × Aminoglycosides* | 0.3 | 0.9 | 0.4 | 0.7 |
| *Flora, fauna × Aminoglycosides* | -5.1 | 6.8 | -0.8 | 0.5 |
| *Human samples × Aminoglycosides* | -1.0 | 1.7 | -0.6 | 0.6 |
| *Human habitat × Aminoglycosides* | -2.8 × 10^2^ | 6.1 × 10^6^ | -4.6 × 10^-5^ | 1.0 |
| *Freshwater × Aminoglycosides* | -5.4 × 10^-3^ | 1.7 × 10^7^ | -3.2 × 10^-10^ | 1.0 |
| *Clinical samples × Aminoglycosides* | -1.7 | 0.9 | -1.8 | 6.9 × 10^-2^ |
| *Sludge, waste × Aminoglycosides* | 0.5 | 3.2 × 10^7^ | 1.5 × 10^-8^ | 1.0 |
| *Soil × Aminoglycosides* | -0.2 | 1.4 | -0.2 | 0.9 |
| *Domestic animals × Trade* | 19.4 | 14.2 | 1.4 | 0.2 |
| *Farms × Trade* | 1.5 | 1.1 | 1.4 | 0.2 |
| *Flora, fauna × Trade* | 3.7 | 4.7 | 0.8 | 0.4 |
| *Human samples × Trade* | 5.1 × 10^-2^ | 1.3 | 3.9 × 10^-2^ | 1.0 |
| *Human habitat × Trade* | 91.7 | 2.3 × 10^6^ | 4.0 × 10^-5^ | 1.0 |
| *Freshwater × Trade* | 0.1 | 1.4 × 10^7^ | 1.0 × 10^-8^ | 1.0 |
| *Clinical samples × Trade* | 0.8 | 1.1 | 0.8 | 0.4 |
| *Sludge, waste × Trade* | -1.1 | 2.9 × 10^7^ | -3.8 × 10^-8^ | 1.0 |
| *Soil × Trade* | -1.6 | 1.8 | -0.8 | 0.4 |
| *Domestic animals × Migration* | -39.8 | 29.7 | -1.3 | 0.2 |
| *Farms × Migration* | -0.8 | 1.1 | -0.7 | 0.5 |
| *Flora, fauna × Migration* | -6.1 | 7.7 | -0.8 | 0.4 |
| *Human samples × Migration* | 0.9 | 1.3 | 0.6 | 0.5 |
| *Human habitat × Migration* | -29.7 | 1.5 × 10^6^ | -2.0 × 10^-5^ | 1.0 |
| *Freshwater × Migration* | 0.7 | 1.8 × 10^7^ | 4.0 × 10^-8^ | 1.0 |
| *Clinical samples × Migration* | 0.8 | 1.1 | 0.7 | 0.5 |
| *Sludge, waste × Migration* | 0.9 | 1.4 × 10^7^ | 6.6 × 10^-8^ | 1.0 |
| *Soil × Migration* | 1.4 | 1.1 | 1.2 | 0.2 |

**Supplementary file 3j: Summary of the selected model for ANTa under high sampling.** *: p<0.05, **: p<0.01, ***: p<0.001.

| Autoregression coefficient φ = -6.9 × 10^-8^, Matérn smoothness ν = 0.3, Matérn scaling ρ = 8.8 | | | | |
| --- | --- | --- | --- | --- |
| **Variable** | **Estimate** | **Conditional standard error** | **t** | **p** |
| *Intercept* | -3.9 | 0.6 | -6.2 | 4.7 × 10^-10^ *** |
| *Domestic animals* | -0.7 | 1.2 | -0.6 | 0.5 |
| *Farms* | -1.5 | 0.7 | -2.0 | 4.5 × 10^-2^ * |
| *Flora, fauna* | -33.2 | 1.1 × 10^7^ | -3.1 × 10^-6^ | 1.0 |
| *Human samples* | 0.3 | 0.5 | 0.5 | 0.6 |
| *Human habitat* | 1.0 | 0.7 | 1.4 | 0.2 |
| *Freshwater* | -33.4 | 1.2 × 10^7^ | -2.8 × 10^-6^ | 1.0 |
| *Clinical samples* | -3.3 | 0.6 | -5.7 | 1.1 × 10^-8^ *** |
| *Sludge, waste* | -1.1 | 1.2 | -0.9 | 0.3 |
| *Soil* | -33.5 | 8.2 × 10^6^ | -4.1 × 10^-6^ | 1.0 |

**Supplementary file 3k: Summary of the selected model for AACh under low sampling.** *: p<0.05, **: p<0.01, ***: p<0.001.

| Autoregression coefficient φ = 9.2 × 10^-7^, Matérn smoothness ν = 16.7, Matérn scaling ρ = 1.6 | | | | |
| --- | --- | --- | --- | --- |
| **Variable** | **Estimate** | **Conditional standard error** | **t** | **p** |
| *Intercept* | -37.9 | 3.9 × 10^6^ | -9.6 × 10^-6^ | 1.0 |
| *Domestic animals* | 33.3 | 3.9 × 10^6^ | 8.4 × 10^-6^ | 1.0 |
| *Farms* | 30.7 | 3.9 × 10^6^ | 7.8 × 10^-6^ | 1.0 |
| *Flora, fauna* | 35.3 | 3.9 × 10^6^ | 9.0 × 10^-6^ | 1.0 |
| *Human samples* | 34.1 | 3.9 × 10^6^ | 8.7 × 10^-6^ | 1.0 |
| *Human habitat* | 34.9 | 3.9 × 10^6^ | 8.8 × 10^-6^ | 1.0 |
| *Freshwater* | -22.2 | 1.3 × 10^7^ | -1.8 × 10^-6^ | 1.0 |
| *Clinical samples* | 33.9 | 3.9 × 10^6^ | 8.6 × 10^-6^ | 1.0 |
| *Sludge, waste* | 35.8 | 3.9 × 10^6^ | 9.1 × 10^-6^ | 1.0 |
| *Soil* | -21.0 | 8.8 × 10^6^ | -2.4 × 10^-6^ | 1.0 |

**Supplementary file 3l: Summary of the selected model for AACh under high sampling.** *: p<0.05, **: p<0.01, ***: p<0.001.

| Autoregression coefficient φ = 0.7, Matérn smoothness ν = 0.6, Matérn scaling ρ = 0.2 | | | | |
| --- | --- | --- | --- | --- |
| **Variable** | **Estimate** | **Conditional standard error** | **t** | **p** |
| *Intercept* | -5.3 | 1.3 | -4.0 | 7.3 × 10^-5^ *** |
| *Domestic animals* | -24.9 | 3.0 × 10^4^ | -8.3 × 10^-4^ | 1.0 |
| *Farms* | 0.4 | 1.3 | 0.3 | 0.7 |
| *Flora, fauna* | -4.3 × 10^2^ | 4.6 × 10^7^ | -9.4 × 10^-6^ | 1.0 |
| *Human samples* | -5.1 | 4.5 | -1.1 | 0.3 |
| *Human habitat* | 5.4 × 10^2^ | 2.3 × 10^5^ | 2.4 × 10^-3^ | 1.0 |
| *Freshwater* | -1.2 × 10^2^ | 1.3 × 10^7^ | -9.9 × 10^-6^ | 1.0 |
| *Clinical samples* | 2.2 | 1.2 | 1.8 | 7.0 × 10^-2^ |
| *Sludge, waste* | 9.7 × 10^2^ | 1.2 × 10^8^ | 8.1 × 10^-6^ | 1.0 |
| *Soil* | -1.2 × 10^2^ | 1.1 × 10^7^ | -1.1 × 10^-5^ | 1.0 |
| *Aminoglycosides* | 0.3 | 0.2 | 1.5 | 0.1 |
| *Trade* | 0.2 | 0.7 | 0.3 | 0.8 |
| *Migration* | 1.0 | 1.4 | 0.7 | 0.5 |
| *Domestic animals × Trade* | 10.1 | 3.1 × 10^4^ | 3.3 × 10^-4^ | 1.0 |
| *Farms × Trade* | 1.4 | 0.9 | 1.7 | 9.7 × 10^-2^ |
| *Flora, fauna × Trade* | 87.2 | 2.0 × 10^7^ | 4.4 × 10^-6^ | 1.0 |
| *Human samples × Trade* | -9.2 | 5.5 | -1.7 | 9.4 × 10^-2^ |
| *Human habitat × Trade* | -44.7 | 3.7 × 10^4^ | -1.2 × 10^-3^ | 1.0 |
| *Freshwater × Trade* | 11.8 | 1.6 × 10^7^ | 7.5 × 10^-7^ | 1.0 |
| *Clinical samples × Trade* | 0.1 | 0.7 | 0.2 | 0.9 |
| *Sludge, waste × Trade* | -1.2 × 10^3^ | 1.2 × 10^8^ | -1.0 × 10^-5^ | 1.0 |
| *Soil × Trade* | 3.9 × 10^-4^ | 1.3 × 10^7^ | 3.0 × 10^-11^ | 1.0 |
| *Domestic animals × Migration* | -2.1 | 5.6 × 10^4^ | -3.8 × 10^-5^ | 1.0 |
| *Farms × Migration* | -2.2 | 1.5 | -1.5 | 0.1 |
| *Flora, fauna × Migration* | -77.9 | 6.1 × 10^7^ | -1.3 × 10^-6^ | 1.0 |
| *Human samples × Migration* | 2.0 | 2.1 | 1.0 | 0.3 |
| *Human habitat × Migration* | -2.8 × 10^2^ | 8.5 × 10^4^ | -3.3 × 10^-3^ | 1.0 |
| *Freshwater × Migration* | -9.7 | 1.4 × 10^7^ | -7.0 × 10^-7^ | 1.0 |
| *Clinical samples × Migration* | -1.6 | 1.4 | -1.1 | 0.3 |
| *Sludge, waste × Migration* | NA | NA | NA | NA |
| *Soil × Migration* | -1.9 | 1.1 × 10^7^ | -1.7 × 10^-7^ | 1.0 |

**Supplementary file 3m: Summary of the selected model for APHf under low sampling.** *: p<0.05, **: p<0.01, ***: p<0.001.

| Autoregression coefficient φ = 2.0 × 10^-7^, Matérn smoothness ν = 0.5, Matérn scaling ρ = 9.3 × 10^-2^ | | | | |
| --- | --- | --- | --- | --- |
| **Variable** | **Estimate** | **Conditional standard error** | **t** | **p** |
| *Intercept* | -5.9 | 1.2 | -4.8 | 1.3 × 10^-6^ *** |
| *Aminoglycosides* | 0.3 | 0.1 | 2.2 | 3.1 × 10^-2^ * |

**Supplementary file 3n: Summary of the selected model for APHf under high sampling.** *: p<0.05, **: p<0.01, ***: p<0.001.

| Autoregression coefficient φ = -0.7, Matérn smoothness ν = 1.1 × 10^-2^, Matérn scaling ρ = 1.0 | | | | |
| --- | --- | --- | --- | --- |
| **Variable** | **Estimate** | **Conditional standard error** | **t** | **p** |
| *Intercept* | -5.8 | 1.9 | -3.0 | 2.4 × 10^-3^ ** |
| *Domestic animals* | -96.9 | 2.9 × 10^7^ | -3.3 × 10^-6^ | 1.0 |
| *Farms* | -1.7 × 10^2^ | 3.5 × 10^6^ | -4.8 × 10^-5^ | 1.0 |
| *Flora, fauna* | -79.6 | 8.4 × 10^7^ | -9.4 × 10^-7^ | 1.0 |
| *Human samples* | -1.4 × 10^2^ | 7.4 × 10^4^ | -1.9 × 10^-3^ | 1.0 |
| *Human habitat* | -4.4 × 10^2^ | 1.8 × 10^7^ | -2.5 × 10^-5^ | 1.0 |
| *Freshwater* | -4.4 × 10^2^ | 1.6 × 10^7^ | -2.7 × 10^-5^ | 1.0 |
| *Clinical samples* | -8.3 | 1.5 | -5.6 | 2.7 × 10^-8^ *** |
| *Sludge, waste* | -3.1 × 10^2^ | 3.7 × 10^7^ | -8.4 × 10^-6^ | 1.0 |
| *Soil* | -1.8 × 10^2^ | 9.2 × 10^6^ | -2.0 × 10^-5^ | 1.0 |
| *Aminoglycosides* | -0.5 | 0.4 | -1.3 | 0.2 |
| *Trade* | -0.7 | 0.4 | -2.1 | 3.8 × 10^-2^ * |
| *Migration* | 3.0 | 1.0 | 3.1 | 2.2 × 10^-3^ ** |
| *Domestic animals × Aminoglycosides* | 3.3 × 10^2^ | 6.5 × 10^7^ | 5.1 × 10^-6^ | 1.0 |
| *Farms × Aminoglycosides* | 61.6 | 6.2 × 10^6^ | 10.0 × 10^-6^ | 1.0 |
| *Flora, fauna × Aminoglycosides* | -3.0 × 10^2^ | 8.7 × 10^7^ | -3.5 × 10^-6^ | 1.0 |
| *Human samples × Aminoglycosides* | -29.8 | 3.8 × 10^4^ | -7.8 × 10^-4^ | 1.0 |
| *Human habitat × Aminoglycosides* | -2.1 × 10^2^ | 6.5 × 10^7^ | -3.2 × 10^-6^ | 1.0 |
| *Freshwater × Aminoglycosides* | 3.2 × 10^2^ | 1.7 × 10^7^ | 1.9 × 10^-5^ | 1.0 |
| *Clinical samples × Aminoglycosides* | 2.7 | 0.7 | 4.0 | 7.7 × 10^-5^ *** |
| *Sludge, waste × Aminoglycosides* | 2.4 × 10^2^ | 1.1 × 10^8^ | 2.2 × 10^-6^ | 1.0 |
| *Soil × Aminoglycosides* | 59.7 | 1.4 × 10^7^ | 4.2 × 10^-6^ | 1.0 |
| *Domestic animals × Trade* | 2.6 × 10^2^ | 7.2 × 10^7^ | 3.6 × 10^-6^ | 1.0 |
| *Farms × Trade* | 9.1 | 4.5 × 10^6^ | 2.0 × 10^-6^ | 1.0 |
| *Flora, fauna × Trade* | -90.5 | 1.8 × 10^7^ | -5.1 × 10^-6^ | 1.0 |
| *Human samples × Trade* | 78.7 | 8.9 × 10^4^ | 8.9 × 10^-4^ | 1.0 |
| *Human habitat × Trade* | 2.0 × 10^2^ | 1.2 × 10^8^ | 1.7 × 10^-6^ | 1.0 |
| *Freshwater × Trade* | -3.2 × 10^2^ | 5.8 × 10^7^ | -5.4 × 10^-6^ | 1.0 |
| *Clinical samples × Trade* | -10.0 | 4.5 | -2.2 | 2.6 × 10^-2^ * |
| *Sludge, waste × Trade* | NA | NA | NA | NA |
| *Soil × Trade* | 67.0 | 4.5 × 10^7^ | 1.5 × 10^-6^ | 1.0 |
| *Domestic animals × Migration* | -96.2 | 2.0 × 10^7^ | -4.7 × 10^-6^ | 1.0 |
| *Farms × Migration* | -12.9 | 7.3 × 10^6^ | -1.8 × 10^-6^ | 1.0 |
| *Flora, fauna × Migration* | NA | NA | NA | NA |
| *Human samples × Migration* | -2.0 × 10^2^ | 1.1 × 10^5^ | -1.7 × 10^-3^ | 1.0 |
| *Human habitat × Migration* | NA | NA | NA | NA |
| *Freshwater × Migration* | 0.5 | 1.7 × 10^7^ | 2.8 × 10^-8^ | 1.0 |
| *Clinical samples × Migration* | -1.2 | 1.3 | -0.9 | 0.4 |
| *Sludge, waste × Migration* | NA | NA | NA | NA |
| *Soil × Migration* | -65.2 | 2.6 × 10^7^ | -2.5 × 10^-6^ | 1.0 |
