# Supplementary material for: Ecology, more than antibiotics consumption, is the major predictor for the global distribution of aminoglycoside-modifying enzymes
Source: eLife. 2023 Feb 14;12:e77015. doi: 10.7554/eLife.77015 (PMC9928423; doi:10.7554/eLife.77015)
Supplement: Supplementary file 4. [file elife-77015-supp4.doc]

**Supplementary file 4a: Summary of the selected model for AACa in biomes where antibiotics are prescribed.** *: p<0.05, **: p<0.01, ***: p<0.001.

| Autoregression coefficient φ = 0.6, Matérn smoothness ν = 3.0 × 10^-2^, Matérn scaling ρ = 9.3 | | | | |
| --- | --- | --- | --- | --- |
| **Variable** | **Estimate** | **Conditional standard error** | **t** | **p** |
| *Intercept* | -38.0 | 1.6 × 10^6^ | -2.4 × 10^-5^ | 1.0 |
| *Human samples* | 0.7 | 2.7 × 10^6^ | 2.5 × 10^-7^ | 1.0 |
| *Clinical samples* | 30.6 | 1.6 × 10^6^ | 1.9 × 10^-5^ | 1.0 |
| *Aminoglycosides* | 0.5 | 0.2 | 2.1 | 3.5 × 10^-2^ * |

**Supplementary file 4b: Summary of the selected model for AACc in biomes where antibiotics are prescribed.** *: p<0.05, **: p<0.01, ***: p<0.001.

| Autoregression coefficient φ = -0.4, Matérn smoothness ν = 1.8, Matérn scaling ρ = 2.4 | | | | |
| --- | --- | --- | --- | --- |
| **Variable** | **Estimate** | **Conditional standard error** | **t** | **p** |
| *Intercept* | -38.3 | 7.1 × 10^2^ | -5.4 × 10^-2^ | 1.0 |
| *Human samples* | 19.5 | 7.1 × 10^2^ | 2.7 × 10^-2^ | 1.0 |
| *Clinical samples* | 22.7 | 7.1 × 10^2^ | 3.2 × 10^-2^ | 1.0 |
| *Aminoglycosides* | 4.3 | 0.4 | 11.4 | 0.0 *** |
| *Trade* | -1.7 | 0.3 | -5.3 | 1.1 × 10^-7^ *** |
| *Migration* | 3.9 | 0.9 | 4.3 | 2.0 × 10^-5^ *** |

**Supplementary file 4c: Summary of the selected model for AACd in biomes where antibiotics are prescribed.** *: p<0.05, **: p<0.01, ***: p<0.001.

| Autoregression coefficient φ = 0.3, Matérn smoothness ν = 16.7, Matérn scaling ρ = 1.1 | | | | |
| --- | --- | --- | --- | --- |
| **Variable** | **Estimate** | **Conditional standard error** | **t** | **p** |
| *Intercept* | -9.1 | 2.1 | -4.4 | 1.1 × 10^-5^ *** |
| *Human samples* | 1.2 | 3.0 | 0.4 | 0.7 |
| *Clinical samples* | 2.9 | 1.9 | 1.5 | 0.1 |
| *Aminoglycosides* | -4.5 | 2.5 | -1.8 | 7.2 × 10^-2^ |
| *Trade* | -4.8 | 3.6 | -1.3 | 0.2 |
| *Migration* | -0.7 | 0.8 | -0.8 | 0.4 |
| *Human samples × Aminoglycosides* | 4.9 | 2.6 | 1.9 | 6.3 × 10^-2^ |
| *Clinical samples × Aminoglycosides* | 4.6 | 2.5 | 1.8 | 6.9 × 10^-2^ |
| *Human samples × Trade* | 3.1 | 4.5 | 0.7 | 0.5 |
| *Clinical samples × Trade* | 5.7 | 3.6 | 1.6 | 0.1 |
| *Human samples × Migration* | -1.9 | 4.7 | -0.4 | 0.7 |
| *Clinical samples × Migration* | -0.3 | 0.9 | -0.3 | 0.7 |

**Supplementary file 4d: Summary of the selected model for AACe1 in biomes where antibiotics are prescribed.** *: p<0.05, **: p<0.01, ***: p<0.001.

| Autoregression coefficient φ = 0.5, Matérn smoothness ν = 0.4, Matérn scaling ρ = 0.1 | | | | |
| --- | --- | --- | --- | --- |
| **Variable** | **Estimate** | **Conditional standard error** | **t** | **p** |
| *Intercept* | -9.5 | 1.4 | -6.8 | 1.3 × 10^-11^ *** |
| *Human samples* | 4.9 | 1.1 | 4.3 | 1.6 × 10^-5^ *** |
| *Clinical samples* | 6.3 | 1.1 | 5.7 | 1.0 × 10^-8^ *** |
| *Aminoglycosides* | 0.3 | 8.7 × 10^-2^ | 2.9 | 3.6 × 10^-3^ ** |
| *Trade* | 3.4 | 0.8 | 4.3 | 1.4 × 10^-5^ *** |
| *Migration* | -4.0 | 1.2 | -3.3 | 8.7 × 10^-4^ *** |
| *Human samples × Trade* | -2.8 | 0.8 | -3.5 | 4.3 × 10^-4^ *** |
| *Clinical samples × Trade* | -3.4 | 0.8 | -4.5 | 8.0 × 10^-6^ *** |
| *Human samples × Migration* | 3.4 | 1.2 | 2.8 | 5.1 × 10^-3^ ** |
| *Clinical samples × Migration* | 3.8 | 1.2 | 3.3 | 1.1 × 10^-3^ ** |

**Supplementary file 4e: Summary of the selected model for AACf1 in biomes where antibiotics are prescribed.** *: p<0.05, **: p<0.01, ***: p<0.001.

| Autoregression coefficient φ = 1.0, Matérn smoothness ν = 16.7, Matérn scaling ρ = 1.9 | | | | |
| --- | --- | --- | --- | --- |
| **Variable** | **Estimate** | **Conditional standard error** | **t** | **p** |
| *Intercept* | -4.5 | 1.6 | -2.8 | 4.6 × 10^-3^ ** |
| *Human samples* | 0.4 | 0.3 | 1.4 | 0.1 |
| *Clinical samples* | -1.3 | 0.2 | -5.7 | 1.4 × 10^-8^ *** |
| *Aminoglycosides* | -0.8 | 0.4 | -2.0 | 4.9 × 10^-2^ * |
| *Trade* | -0.9 | 0.3 | -3.1 | 2.0 × 10^-3^ ** |
| *Migration* | 1.0 | 0.3 | 3.4 | 7.2 × 10^-4^ *** |
| *Human samples × Aminoglycosides* | -0.7 | 0.6 | -1.3 | 0.2 |
| *Clinical samples × Aminoglycosides* | 1.3 | 0.4 | 3.1 | 1.7 × 10^-3^ ** |
| *Human samples × Trade* | 0.5 | 0.2 | 2.2 | 2.8 × 10^-2^ * |
| *Clinical samples × Trade* | 0.6 | 0.2 | 3.2 | 1.1 × 10^-3^ ** |
| *Human samples × Migration* | -5.5 × 10^-3^ | 0.3 | -1.9 × 10^-2^ | 1.0 |
| *Clinical samples × Migration* | -0.7 | 0.3 | -2.6 | 8.9 × 10^-3^ ** |

**Supplementary file 4f: Summary of the selected model for AACg in biomes where antibiotics are prescribed.** *: p<0.05, **: p<0.01, ***: p<0.001.

| Autoregression coefficient φ = 0.4, Matérn smoothness ν = 16.7, Matérn scaling ρ = 1.9 | | | | |
| --- | --- | --- | --- | --- |
| **Variable** | **Estimate** | **Conditional standard error** | **t** | **p** |
| *Intercept* | -5.3 | 0.5 | -10.0 | 0.0 *** |
| *Human samples* | 1.1 | 0.5 | 2.4 | 1.7 × 10^-2^ * |
| *Clinical samples* | -0.2 | 0.4 | -0.6 | 0.6 |
| *Aminoglycosides* | 0.3 | 0.5 | 0.6 | 0.5 |
| *Trade* | 0.3 | 0.2 | 1.6 | 0.1 |
| *Migration* | -0.5 | 0.3 | -1.6 | 0.1 |
| *Human samples × Aminoglycosides* | -1.4 | 0.7 | -2.1 | 3.3 × 10^-2^ * |
| *Clinical samples × Aminoglycosides* | -0.7 | 0.5 | -1.5 | 0.1 |
| *Human samples × Trade* | -3.8 × 10^-2^ | 0.2 | -0.2 | 0.9 |
| *Clinical samples × Trade* | -0.3 | 0.2 | -1.7 | 9.1 × 10^-2^ |
| *Human samples × Migration* | 0.4 | 0.3 | 1.2 | 0.2 |
| *Clinical samples × Migration* | 1.5 | 0.3 | 5.1 | 3.8 × 10^-7^ *** |

**Supplementary file 4g: Summary of the selected model for ANTa in biomes where antibiotics are prescribed.** *: p<0.05, **: p<0.01, ***: p<0.001.

| Autoregression coefficient φ = 0.5, Matérn smoothness ν = 16.7, Matérn scaling ρ = 1.2 | | | | |
| --- | --- | --- | --- | --- |
| **Variable** | **Estimate** | **Conditional standard error** | **t** | **p** |
| *Intercept* | -5.8 | 0.7 | -8.4 | 1.1 × 10^-16^ *** |
| *Human samples* | 1.5 | 0.4 | 3.5 | 4.3 × 10^-4^ *** |
| *Clinical samples* | -0.2 | 0.4 | -0.4 | 0.7 |
| *Aminoglycosides* | 0.7 | 0.4 | 1.9 | 6.3 × 10^-2^ |
| *Trade* | 1.0 | 0.4 | 2.4 | 1.5 × 10^-2^ * |
| *Migration* | -1.6 | 0.6 | -2.5 | 1.3 × 10^-2^ * |
| *Human samples × Aminoglycosides* | -0.6 | 0.5 | -1.2 | 0.2 |
| *Clinical samples × Aminoglycosides* | -1.1 | 0.4 | -2.4 | 1.5 × 10^-2^ * |
| *Human samples × Trade* | -0.7 | 0.4 | -1.7 | 8.1 × 10^-2^ |
| *Clinical samples × Trade* | -0.9 | 0.4 | -2.2 | 3.0 × 10^-2^ * |
| *Human samples × Migration* | 1.4 | 0.6 | 2.1 | 3.5 × 10^-2^ * |
| *Clinical samples × Migration* | 2.4 | 0.6 | 3.9 | 8.3 × 10^-5^ *** |

**Supplementary file 4h: Summary of the selected model for ANTb in biomes where antibiotics are prescribed.** *: p<0.05, **: p<0.01, ***: p<0.001.

| Autoregression coefficient φ = -0.3, Matérn smoothness ν = 16.7, Matérn scaling ρ = 1.8 | | | | |
| --- | --- | --- | --- | --- |
| **Variable** | **Estimate** | **Conditional standard error** | **t** | **p** |
| *Intercept* | -7.1 | 0.7 | -9.9 | 0.0 *** |
| *Human samples* | 2.7 | 0.6 | 4.3 | 1.6 × 10^-5^ *** |
| *Clinical samples* | 1.0 | 0.6 | 1.7 | 9.0 × 10^-2^ |
| *Trade* | 1.4 | 0.5 | 3.1 | 2.2 × 10^-3^ ** |
| *Migration* | -1.1 | 0.8 | -1.5 | 0.1 |
| *Human samples × Trade* | -1.1 | 0.5 | -2.3 | 2.1 × 10^-2^ * |
| *Clinical samples × Trade* | -0.7 | 0.5 | -1.5 | 0.1 |
| *Human samples × Migration* | 1.0 | 0.7 | 1.3 | 0.2 |
| *Clinical samples × Migration* | 0.7 | 0.7 | 0.9 | 0.4 |

**Supplementary file 4i: Summary of the selected model for ANTd in biomes where antibiotics are prescribed.** *: p<0.05, **: p<0.01, ***: p<0.001.

| Autoregression coefficient φ = -0.1, Matérn smoothness ν = 16.7, Matérn scaling ρ = 3.1 | | | | |
| --- | --- | --- | --- | --- |
| **Variable** | **Estimate** | **Conditional standard error** | **t** | **p** |
| *Intercept* | -2.1 × 10^3^ | 2.1 × 10^5^ | -1.0 × 10^-2^ | 1.0 |
| *Human samples* | 2.1 × 10^3^ | 2.1 × 10^5^ | 1.0 × 10^-2^ | 1.0 |
| *Clinical samples* | 2.1 × 10^3^ | 2.1 × 10^5^ | 1.0 × 10^-2^ | 1.0 |
| *Trade* | 5.3 × 10^2^ | 5.5 × 10^4^ | 9.6 × 10^-3^ | 1.0 |
| *Migration* | -2.7 × 10^3^ | 2.7 × 10^5^ | -1.0 × 10^-2^ | 1.0 |
| *Human samples × Trade* | -5.3 × 10^2^ | 5.5 × 10^4^ | -9.6 × 10^-3^ | 1.0 |
| *Clinical samples × Trade* | -5.3 × 10^2^ | 5.5 × 10^4^ | -9.6 × 10^-3^ | 1.0 |
| *Human samples × Migration* | 2.7 × 10^3^ | 2.7 × 10^5^ | 1.0 × 10^-2^ | 1.0 |
| *Clinical samples × Migration* | 2.7 × 10^3^ | 2.7 × 10^5^ | 1.0 × 10^-2^ | 1.0 |

**Supplementary file 4j: Summary of the selected model for APHa in biomes where antibiotics are prescribed.** *: p<0.05, **: p<0.01, ***: p<0.001.

| Autoregression coefficient φ = 7.9 × 10^-11^, Matérn smoothness ν = 1.7 × 10^-2^, Matérn scaling ρ = 12.4 | | | | |
| --- | --- | --- | --- | --- |
| **Variable** | **Estimate** | **Conditional standard error** | **t** | **p** |
| *Intercept* | -6.0 | 0.5 | -11.3 | 0.0 *** |
| *Human samples* | -31.0 | 2.0 × 10^6^ | -1.6 × 10^-5^ | 1.0 |
| *Clinical samples* | -1.5 | 0.7 | -2.0 | 4.0 × 10^-2^ * |
| *Trade* | 1.0 | 0.2 | 5.6 | 2.1 × 10^-8^ *** |
| *Migration* | 0.4 | 0.2 | 1.6 | 9.9 × 10^-2^ |
| *Human samples × Trade* | -1.0 | 1.7 × 10^6^ | -5.5 × 10^-7^ | 1.0 |
| *Clinical samples × Trade* | -0.2 | 0.3 | -0.6 | 0.5 |
| *Human samples × Migration* | -0.4 | 2.5 × 10^6^ | -1.5 × 10^-7^ | 1.0 |
| *Clinical samples × Migration* | -1.8 | 0.9 | -2.0 | 4.6 × 10^-2^ * |

**Supplementary file 4k: Summary of the selected model for APHd1 in biomes where antibiotics are prescribed.** *: p<0.05, **: p<0.01, ***: p<0.001.

| Autoregression coefficient φ = 3.4 × 10^-7^, Matérn smoothness ν = 16.7, Matérn scaling ρ = 3.5 | | | | |
| --- | --- | --- | --- | --- |
| **Variable** | **Estimate** | **Conditional standard error** | **t** | **p** |
| *Intercept* | -39.9 | 1.6 × 10^6^ | -2.5 × 10^-5^ | 1.0 |
| *Human samples* | 29.4 | 1.6 × 10^6^ | 1.8 × 10^-5^ | 1.0 |
| *Clinical samples* | 31.7 | 1.6 × 10^6^ | 2.0 × 10^-5^ | 1.0 |
| *Aminoglycosides* | -3.8 | 1.4 | -2.8 | 4.7 × 10^-3^ ** |
| *Trade* | 0.3 | 0.3 | 1.0 | 0.3 |
| *Migration* | 0.6 | 0.4 | 1.4 | 0.2 |

**Supplementary file 4l: Summary of the selected model for AACh in biomes where antibiotics are prescribed.** *: p<0.05, **: p<0.01, ***: p<0.001.

| Autoregression coefficient φ = 0.9, Matérn smoothness ν = 16.7, Matérn scaling ρ = 1.5 | | | | |
| --- | --- | --- | --- | --- |
| **Variable** | **Estimate** | **Conditional standard error** | **t** | **p** |
| *Intercept* | -5.6 | 0.8 | -7.1 | 9.8 × 10^-13^ *** |
| *Human samples* | 0.5 | 0.6 | 0.8 | 0.5 |
| *Clinical samples* | 1.9 | 0.4 | 4.5 | 6.9 × 10^-6^ *** |
| *Trade* | 0.6 | 0.3 | 1.9 | 5.1 × 10^-2^ |
| *Migration* | -1.1 | 0.5 | -2.2 | 3.1 × 10^-2^ * |
| *Human samples × Trade* | -8.4 × 10^-2^ | 0.4 | -0.2 | 0.8 |
| *Clinical samples × Trade* | -0.7 | 0.3 | -2.2 | 2.8 × 10^-2^ * |
| *Human samples × Migration* | 1.4 | 0.6 | 2.2 | 2.8 × 10^-2^ * |
| *Clinical samples × Migration* | 0.8 | 0.5 | 1.6 | 0.1 |

**Supplementary file 4m: Summary of the selected model for AACi in biomes where antibiotics are prescribed.** *: p<0.05, **: p<0.01, ***: p<0.001.

| Autoregression coefficient φ = 0.9, Matérn smoothness ν = 5.0 × 10^-3^, Matérn scaling ρ = 6.9 | | | | |
| --- | --- | --- | --- | --- |
| **Variable** | **Estimate** | **Conditional standard error** | **t** | **p** |
| *Intercept* | -6.5 | 0.7 | -9.2 | 0.0 *** |
| *Human samples* | -30.3 | 2.0 × 10^6^ | -1.5 × 10^-5^ | 1.0 |
| *Clinical samples* | -1.0 | 0.7 | -1.5 | 0.1 |
| *Aminoglycosides* | -0.5 | 0.6 | -0.8 | 0.4 |
| *Trade* | 1.1 | 0.2 | 5.0 | 4.8 × 10^-7^ *** |
| *Migration* | 0.2 | 0.4 | 0.4 | 0.7 |
| *Human samples × Trade* | -1.2 | 1.6 × 10^6^ | -7.2 × 10^-7^ | 1.0 |
| *Clinical samples × Trade* | -0.6 | 0.3 | -1.7 | 8.8 × 10^-2^ |
| *Human samples × Migration* | -0.3 | 2.5 × 10^6^ | -1.3 × 10^-7^ | 1.0 |
| *Clinical samples × Migration* | -0.9 | 0.8 | -1.1 | 0.3 |

**Supplementary file 4n: Summary of the selected model for AACj in biomes where antibiotics are prescribed.** *: p<0.05, **: p<0.01, ***: p<0.001.

| Autoregression coefficient φ = -0.7, Matérn smoothness ν = 16.7, Matérn scaling ρ = 4.2 | | | | |
| --- | --- | --- | --- | --- |
| **Variable** | **Estimate** | **Conditional standard error** | **t** | **p** |
| *Intercept* | -38.5 | 7.9 × 10^5^ | -4.9 × 10^-5^ | 1.0 |
| *Human samples* | 26.5 | 7.9 × 10^5^ | 3.4 × 10^-5^ | 1.0 |
| *Clinical samples* | 29.3 | 7.9 × 10^5^ | 3.7 × 10^-5^ | 1.0 |
| *Trade* | 1.0 | 0.4 | 2.5 | 1.1 × 10^-2^ * |
| *Migration* | -0.4 | 0.3 | -1.4 | 0.2 |

**Supplementary file 4o: Summary of the selected model for APHf in biomes where antibiotics are prescribed.** *: p<0.05, **: p<0.01, ***: p<0.001.

| Autoregression coefficient φ = 6.8 × 10^-8^, Matérn smoothness ν = 1.3 × 10^-2^, Matérn scaling ρ = 9.0 | | | | |
| --- | --- | --- | --- | --- |
| **Variable** | **Estimate** | **Conditional standard error** | **t** | **p** |
| *Intercept* | -11.2 | 2.0 | -5.5 | 4.2 × 10^-8^ *** |
| *Human samples* | 4.8 | 2.0 | 2.4 | 1.6 × 10^-2^ * |
| *Clinical samples* | 5.3 | 1.9 | 2.7 | 6.1 × 10^-3^ ** |
| *Trade* | 2.2 | 0.8 | 2.6 | 8.3 × 10^-3^ ** |
| *Migration* | -6.7 | 2.9 | -2.3 | 2.2 × 10^-2^ * |
| *Human samples × Trade* | -2.3 | 1.2 | -1.9 | 5.5 × 10^-2^ |
| *Clinical samples × Trade* | -2.8 | 0.9 | -3.2 | 1.4 × 10^-3^ ** |
| *Human samples × Migration* | 5.5 | 2.9 | 1.9 | 5.8 × 10^-2^ |
| *Clinical samples × Migration* | 6.8 | 2.9 | 2.4 | 1.9 × 10^-2^ * |
