# Supplementary material for: Ecology, more than antibiotics consumption, is the major predictor for the global distribution of aminoglycoside-modifying enzymes
Source: eLife. 2023 Feb 14;12:e77015. doi: 10.7554/eLife.77015 (PMC9928423; doi:10.7554/eLife.77015)
Supplement: Supplementary file 5. [file elife-77015-supp5.doc]

**Supplementary file 5: Human exchanges variables influencing the prevalence of ACBs**

|  | AACb | AACc | AACe1 | AACf1 | AACg | ANTa | ANTb | APHa | APHd1 | AACh | AACi | AACj | APHf |
| --- | --- | --- | --- | --- | --- | --- | --- | --- | --- | --- | --- | --- | --- |
| Animal and vegetable oils, fats and waxes |  |  |  |  |  |  |  | *×* | *×* |  |  | *×* |  |
| Beverages | *×* |  |  |  |  |  |  | *×* | *×* |  | *×* | *×* |  |
| Cereals and cereal preparations |  |  |  |  |  |  |  | *×* | *×* | *×* | *×* |  |  |
| Coal, coke and briquettes |  |  |  |  |  |  |  | *×* | *×* |  | *×* |  |  |
| Coffee, tea, cocoa, spices, and manufactures thereof |  |  |  |  |  |  | *×* | *×* | *×* |  |  |  |  |
| Cork and wood |  |  |  |  |  |  |  | *×* | *×* |  | *×* |  |  |
| Crude materials, inedible, except fuels |  |  | *×* |  |  |  | *×* |  | *×* |  |  |  |  |
| Crude rubber (including synthetic and reclaimed) |  |  | *×* |  |  |  |  |  |  |  |  | *×* |  |
| Dairy products and birds' eggs |  |  |  |  |  |  |  | *×* | *×* |  |  |  |  |
| Dyeing, tanning and colouring materials |  |  |  |  |  |  |  | *×* | *×* |  |  | *×* |  |
| Feedstuff for animals (excluding unmilled cereals) |  |  |  |  | *×* |  |  | *×* | *×* |  | *×* |  |  |
| Fish, crustaceans, molluscs and preparations thereof |  |  |  |  |  |  |  | *×* | *×* | *×* |  |  |  |
| Food and live animals |  |  |  |  |  |  |  | *×* | *×* |  | *×* |  |  |
| Hides, skins and furskins, raw |  |  |  |  |  |  |  |  | *×* | *×* |  |  |  |
| Live animals other than animals of division 03 |  |  |  |  |  |  |  |  | *×* |  |  |  |  |
| Manufactured goods |  |  |  |  |  |  |  | *×* | *×* |  | *×* |  |  |
| Meat and meat preparations |  |  |  |  |  |  |  | *×* | *×* | *×* |  |  |  |
| Medicinal and pharmaceutical products |  |  |  |  |  | *×* |  | *×* | *×* | *×* |  | *×* |  |
| Mineral fuels, lubricants and related materials |  |  |  |  |  |  |  | *×* | *×* |  |  |  |  |
| Miscellaneous edible products and preparations | *×* |  |  |  |  |  |  | *×* | *×* |  |  | *×* |  |
| Oil seeds and oleaginous fruits |  |  |  | *×* |  |  |  |  | *×* |  |  | *×* |  |
| Petroleum, petroleum products and related materials | *×* |  |  |  |  |  |  | *×* | *×* |  |  |  |  |
| Plastics |  |  |  |  |  |  |  | *×* | *×* |  |  | *×* |  |
| Sugar, sugar preparations and honey |  |  |  |  |  | *×* |  | *×* | *×* | *×* |  |  |  |
| Textiles fibres and their wastes |  |  | *×* |  |  |  |  |  | *×* |  |  |  |  |
| Tobacco and tobacco manufactures |  | *×* |  |  |  |  |  |  |  |  |  |  |  |
| Vegetables and fruits |  |  |  |  |  |  |  | *×* | *×* |  | *×* |  |  |
| Human migration |  |  |  |  |  | *×* |  |  | *×* |  |  |  | *×* |
