# Supplementary material for: Ecology, more than antibiotics consumption, is the major predictor for the global distribution of aminoglycoside-modifying enzymes
Source: eLife. 2023 Feb 14;12:e77015. doi: 10.7554/eLife.77015 (PMC9928423; doi:10.7554/eLife.77015)
Supplement: Supplementary file 6. [file elife-77015-supp6.doc]

**Supplementary file 6: Classification of biomes according to the keywords of BioSamples.** The “order” column refers to the order in which sampling contexts were assigned to each biome. Once assigned to one biome, sampling contexts could not be assigned to any other biome.

| **Biome** | **Order** | **Keywords** |
| --- | --- | --- |
| Clinical | 1 | - “hospit”, “clinic”, or “medical” - “human” or “homo” and at least one of the following keywords: “disease”, “blood”, “oral”, “feces”, “infection”, “arthr”, “hemo”, “bronch”, “pulmo”, “respi”, “urine”, “failure”, “pus”, “throat”, “thora”, “semen”, “wound”, “skin”, “gut”, “intestin”, “septicemia”, “sputum”, “fibrosis”, “swab”, “fluid”, “itis”, “bile”, “pneumonia”, “sputamentum”, “sick”, “gastr”, “aspirate”, “fecal”, “sudate”, “groin”, “emia”, “faecal”, “nares”, “osis”, “excreted”, “sepsis”, “patient”, “vagin”, “rect”, “surg”, “drainage”, “trach”, “lung”, “nasal”, “tissue”, “cornea”, “nosocomial”, “ICU”, “fever”, “bronc”, “absc”, “phary” - host not specified but any of the keywords: “melioidosis”, “tract infection”, “burn”, “urine”, “feces”, “blood”, “stool”, “sputum” |
| Human habitat | 2 | “habitat”, “rural”, “bath”, “potable”, “tap water”, “toilet”, “spacecraft”, “food” |
| Domestic animals | 3 | “canis”, “canine”, “feline”, “dog” (but not “hot-dog” or “prairie dog”), “felis”, “rattus”, “cavia”, “cricet”, “chinchilla”, “mustela”, “mus”, “serinus”, “tortoise”, “parrot”, “carassius”, “mouse” |
| Farms | 4 | “bos”, “livestock”, “ovis”, “ovine”, “sheep”, “capra”, “meat”, “anas”, “goose”, “duck”, “cow”, “ovine”, “fish farm”, “slaughterhouse”, “poultry”, “gallus”, “pig”, “pork”, “beef”, “meleagris”, “oyster” (if also containing “cult”), “mellifera”, “milk”, “hen”, “chicken”, “lamb”, “egg”, “cattle”, “turkey”, “swine”, “porcine”, “goat”, “equine”, “horse”, “rabbit”, “calf”, “sus scrofa”, “equus”, “oryctolagus”, “manure”, “dairy”, “chease”, “sus” (if also containing “disease”), “farm” |
| Agrosystems | 5 | “wheat”, “salad”, “triticum”, “maize”, “zea mays”, “rice”, “oryza”, “field”, “plantation”, “olive”, “coffee”, “coffea”, “bean”, “banana”, “tomato”, “potato”, “solanum”, “musa”, “avena”, “oats”, “soy”, “brassica”, “hordeum”, “barley”, “rye”, “sorghum”, “millet”, “phaseolus”, “saccharum, “quinoa”, “cicer”, “pisum”, “sesam”, “cassava”, “sugar”, “manihot”, “ipomo”, “coconut”, “cotton”, “oil palm”, “helianthus”, “berry”, “onion”, “grape”, “walnut”, “peanut”, “prunus”, “citrus”, “greenhouse”, “lentil”, “tea”, “camellia”, “compost”, “tobacco”, “nicotiana”, “lettuce”, “vegetable”, “carrot”, “cucumber”, “pepper”, “pea”, “cilantro” |
| Wild plants and animals | 6 | “tapir”, “panthera”, “forest”, “grass”, “prairie dog”, “snake”, “ursus”, “ursi”, “migratory”, “crow”, “pika”, “gull”, “bird”, “clover”, “grazing”, “monkey”, “elephant”, “corvus”, “zebra”, “lily”, “vulture” |
| Freshwater | 7 | “river”, “marsh”, “pond”, “lake”, “swamp”, “bog”, “glacier”, “freshwater”, “fresh water”, “aquifer”, “permafrost”, “ground water”, “wetland”, “spring”, “catfish”, “creek”, “mineral water”, “environmental (streams)” |
| Sea water | 8 | “ocean”, “seawater”, “sea”, “sea water”, “hydrotherm”, “continental shelf”, “saltwater”, “salt water”, “algae”, “tidal”, “tuna”, “seafood”, “shrimp” |
| Sludge, waste | 9 | “wastewater”, “waste water”, “sewage”, “sludge” |
| Soil | 10 | “soil”, “terrestrial”, “sand”, “humus” |
| Human | 11 | “homo sapiens” or “human” with no other specification |
