# Supplementary material for: Ecology, more than antibiotics consumption, is the major predictor for the global distribution of aminoglycoside-modifying enzymes
Source: eLife. 2023 Feb 14;12:e77015. doi: 10.7554/eLife.77015 (PMC9928423; doi:10.7554/eLife.77015)
Supplement: Supplementary file 7. [file elife-77015-supp7.doc]

**Supplementary file 7a: Impact of the association of AME genes with mobile genetic elements with their duplication risk**

| **Minimum identity between genomic contexts** | **Minimum identity between genes** | **Odds ratio** | **500-permutation p-value** |
| --- | --- | --- | --- |
| 50% | 80% | 14.2 | 0.001 |
| 50% | 85% | 11.9 | 0.001 |
| 50% | 90% | 10.7 | 0.001 |
| 50% | 95% | 10.4 | 0.001 |
| 70% | 80% | 14.2 | 0.001 |
| 70% | 85% | 11.9 | 0.001 |
| 70% | 90% | 10.7 | 0.001 |
| 70% | 95% | 10.4 | 0.001 |
| 80% | 80% | 14.2 | 0.001 |
| 0.001 | 85% | 11.9 | 0.001 |
| 0.001 | 90% | 10.7 | 0.001 |
| 80% | 95% | 10.4 | 0.001 |
| 90% | 80% | 14.2 | 0.001 |
| 90% | 85% | 11.9 | 0.001 |
| 90% | 90% | 10.7 | 0.001 |
| 90% | 95% | 10.4 | 0.001 |

**Supplementary file 7b: Impact of the association of AME genes with different types of mobile genetic elements with their duplication risk**

| **Minimum identity between genomic contexts** | **Minimum identity between genes** | **Type of mobile genetic element** | **Odds ratio** | **500-permutation p-value** |
| --- | --- | --- | --- | --- |
| 50% | 80% | Intergenomic mobility | 18.5 | 0.001 |
| 50% | 85% | Intergenomic mobility | 14.7 | 0.001 |
| 50% | 90% | Intergenomic mobility | 13.7 | 0.001 |
| 50% | 95% | Intergenomic mobility | 13.3 | 0.001 |
| 70% | 80% | Intergenomic mobility | 18.5 | 0.001 |
| 70% | 85% | Intergenomic mobility | 14.7 | 0.001 |
| 70% | 90% | Intergenomic mobility | 13.7 | 0.001 |
| 70% | 95% | Intergenomic mobility | 13.3 | 0.001 |
| 80% | 80% | Intergenomic mobility | 18.5 | 0.001 |
| 80% | 85% | Intergenomic mobility | 14.7 | 0.001 |
| 80% | 90% | Intergenomic mobility | 13.7 | 0.001 |
| 80% | 95% | Intergenomic mobility | 13.3 | 0.001 |
| 90% | 80% | Intergenomic mobility | 18.5 | 0.001 |
| 90% | 85% | Intergenomic mobility | 14.7 | 0.001 |
| 90% | 90% | Intergenomic mobility | 13.7 | 0.001 |
| 90% | 95% | Intergenomic mobility | 13.3 | 0.001 |
| 50% | 80% | Intragenomic mobility | 12.0 | 0.001 |
| 50% | 85% | Intragenomic mobility | 12.0 | 0.002 |
| 50% | 90% | Intragenomic mobility | 12.0 | 0.002 |
| 50% | 95% | Intragenomic mobility | 10.8 | 0.003 |
| 70% | 80% | Intragenomic mobility | 12.0 | 0.001 |
| 70% | 85% | Intragenomic mobility | 12.0 | 0.002 |
| 70% | 90% | Intragenomic mobility | 12.0 | 0.002 |
| 70% | 95% | Intragenomic mobility | 10.8 | 0.003 |
| 80% | 80% | Intragenomic mobility | 12.0 | 0.001 |
| 80% | 85% | Intragenomic mobility | 12.0 | 0.002 |
| 80% | 90% | Intragenomic mobility | 12.0 | 0.002 |
| 80% | 95% | Intragenomic mobility | 10.8 | 0.003 |
| 90% | 80% | Intragenomic mobility | 12.0 | 0.001 |
| 90% | 85% | Intragenomic mobility | 12.0 | 0.002 |
| 90% | 90% | Intragenomic mobility | 12.0 | 0.002 |
| 90% | 95% | Intragenomic mobility | 10.8 | 0.003 |
| 50% | 80% | Intra- and intergenomic mobility | 16.0 | 0.001 |
| 50% | 85% | Intra- and intergenomic mobility | 14.1 | 0.001 |
| 50% | 90% | Intra- and intergenomic mobility | 12.1 | 0.001 |
| 50% | 95% | Intra- and intergenomic mobility | 12.1 | 0.001 |
| 70% | 80% | Intra- and intergenomic mobility | 16.0 | 0.001 |
| 70% | 85% | Intra- and intergenomic mobility | 14.1 | 0.001 |
| 70% | 90% | Intra- and intergenomic mobility | 12.1 | 0.001 |
| 70% | 95% | Intra- and intergenomic mobility | 12.1 | 0.001 |
| 80% | 80% | Intra- and intergenomic mobility | 16.0 | 0.001 |
| 80% | 85% | Intra- and intergenomic mobility | 14.1 | 0.001 |
| 80% | 90% | Intra- and intergenomic mobility | 12.1 | 0.001 |
| 80% | 95% | Intra- and intergenomic mobility | 12.1 | 0.001 |
| 90% | 80% | Intra- and intergenomic mobility | 16.0 | 0.001 |
| 90% | 85% | Intra- and intergenomic mobility | 14.1 | 0.001 |
| 90% | 90% | Intra- and intergenomic mobility | 12.1 | 0.001 |
| 90% | 95% | Intra- and intergenomic mobility | 12.1 | 0.001 |
